# Cyclotraxin-B, the First Highly Potent and Selective TrkB Inhibitor, Has Anxiolytic Properties in Mice

**DOI:** 10.1371/journal.pone.0009777

**Published:** 2010-03-19

**Authors:** Maxime Cazorla, Anne Jouvenceau, Christiane Rose, Jean-Philippe Guilloux, Catherine Pilon, Alex Dranovsky, Joël Prémont

**Affiliations:** 1 Neurobiology & Molecular Pharmacology, Centre de Psychiatrie et de Neurosciences, UMR-894 INSERM/Université Paris Descartes, Paris, France; 2 Pathophysiology of Ageing Brain, Centre de Psychiatrie et de Neurosciences, UMR-894 INSERM/Université Paris Descartes, Paris, France; 3 Department of Psychiatry, Columbia University, New York State Psychiatric Institute, New York, New York, United States of America; RIKEN Brain Science Institution, Japan

## Abstract

In the last decades, few mechanistically novel therapeutic agents have been developed to treat mental and neurodegenerative disorders. Numerous studies suggest that targeting BDNF and its TrkB receptor could be a promising therapeutic strategy for the treatment of brain disorders. However, the development of potent small ligands for the TrkB receptor has proven to be difficult. By using a peptidomimetic approach, we developed a highly potent and selective TrkB inhibitor, cyclotraxin-B, capable of altering TrkB-dependent molecular and physiological processes such as synaptic plasticity, neuronal differentiation and BDNF-induced neurotoxicity. Cyclotraxin-B allosterically alters the conformation of TrkB, which leads to the inhibition of both BDNF-dependent and -independent (basal) activities. Finally, systemic administration of cyclotraxin-B to mice results in TrkB inhibition in the brain with specific anxiolytic-like behavioral effects and no antidepressant-like activity. This study demonstrates that cyclotraxin-B might not only be a powerful tool to investigate the role of BDNF and TrkB in physiology and pathology, but also represents a lead compound for the development of new therapeutic strategies to treat brain disorders.

## Introduction

Brain-Derived Neurotrophic Factor (BDNF) belongs to the neurotrophin family that regulates neuronal development and survival by interacting with two classes of cell surface receptors, TrkB receptor and the non-selective p75^NTR^ receptor [1]. Binding of BDNF to TrkB triggers receptor dimerization and subsequent autophosphorylation on tyrosine residues. In addition, TrkB receptors can be activated in absence of BDNF either through spontaneous dimerizations or through different signal transduction systems, including dopamine, adenosine, Pituitary Adenylate Cyclase-Activating Polypeptides (PACAP), endocannabinoids, glucocorticoids or the inorganic ion Zinc [2], [3], [4], [5], [6], [7]. Although BDNF was initially considered to be involved in the development and maintenance of central and peripheral nervous systems, more recent evidence have implicated BDNF in the regulation of synaptic strength and long-term memory processes [8].

Given its trophic effects on neurons and its central role in high-order cognitive functions, BDNF has rapidly emerged as a key element in the pathophysiology of numerous brain disorders, including neurological disorders [*e.g.* epileptogenesis [9]], neurodegenerative diseases [*e.g.* amyotrophic lateral sclerosis [10], Huntington [11], Alzheimer's and Parkinson's diseases [12]] and psychiatric disorders [*e.g.* anxiety/depression [13], [14], addiction [15] and schizophrenic psychosis [16]]. Altogether, these observations present BDNF and TrkB as a promising new therapeutic target. However, due to the lack of specific modulators, the behavioral consequences of a systemic intervention on the BDNF/TrkB system in these pathologies and in high-order cognitive functions still remain elusive.

Since no structural data are available for the BDNF/TrkB complex, the development of specific ligands has been difficult to address. Moreover, the large surface of the putative binding domain for BDNF makes the design of small molecules more complex. Numerous studies have implicated the solvent-exposed loops of BDNF in mediating their biological effects. Site-directed mutagenesis analyses, production of chimeric neurotrophins and mimetic peptides have highlighted specific and variable regions among neurotrophins that are important for the binding specificity and/or activation of their cognate Trk receptors [for reviews, see [17], [18], [19]]. Moreover, other groups have designed functionally active peptidomimetics of neurotrophins [see examples in [20], [21], [22], [23], [24], [25], [26], [27]], demonstrating the feasibility of this strategy. Therefore, to develop a potent TrkB ligand active *in vivo*, we took advantage of these seminal studies and we designed several BDNF-derived mimetic peptides, which were tested for their ability to modulate the activity of the human TrkB receptor. Here, we describe the properties of a small inhibitor peptide mimicking the reverse turn structure of the variable region III that protrudes from the core of BDNF, which we named cyclotraxin-B.

## Results

### Strategy for the design of cyclotraxin-B

**Figure 1:**
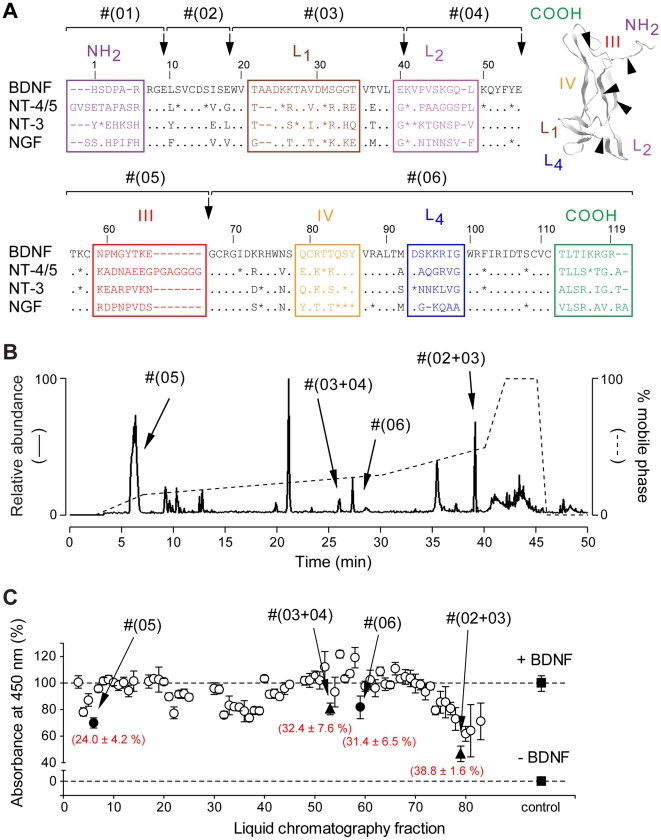
Purification and identification of binding determinants of BDNF. (**A**) Alignment of the amino-acid sequences of rat BDNF, NT-4/5, NT-3 and NGF (numbered based on BDNF sequence, dashes represent gaps, points represent identities and stars represent strongly similar amino acids). Variable regions are boxed and labeled (Loops L1, L2, L3 and L4). (***inset***) Three-dimensional structure of a BDNF monomer (PDB entry, *1bnd*). Isolation of these regions by endoproteinase Glu-C V8 (black arrows) resulted in the production of six fragments (#(01) to #(06)). (**B**) Fragments were purified by HPLC using a non-linear gradient (dashed line) and identified by ESI-MS (solid line). All expected fragments and fragments resulting from miscleavage of BDNF were found. Only the four fragments capable of inhibiting the BDNF-induced TrkB activity in (**C**) are noted. (**C**) Representative KIRA-ELISA inhibition profile of 80 HPLC fractions. Fractions (∼0.3 µM final) were assayed in the presence of 1 nM BDNF. Most fractions did not produce significant inhibitions (○) except four fractions (• single fragments, ▴ miscleavage fragments). Mean ± s.e.m. of values obtained in triplicate in 8 independent experiments are noted in brackets.

**Figure 2:**
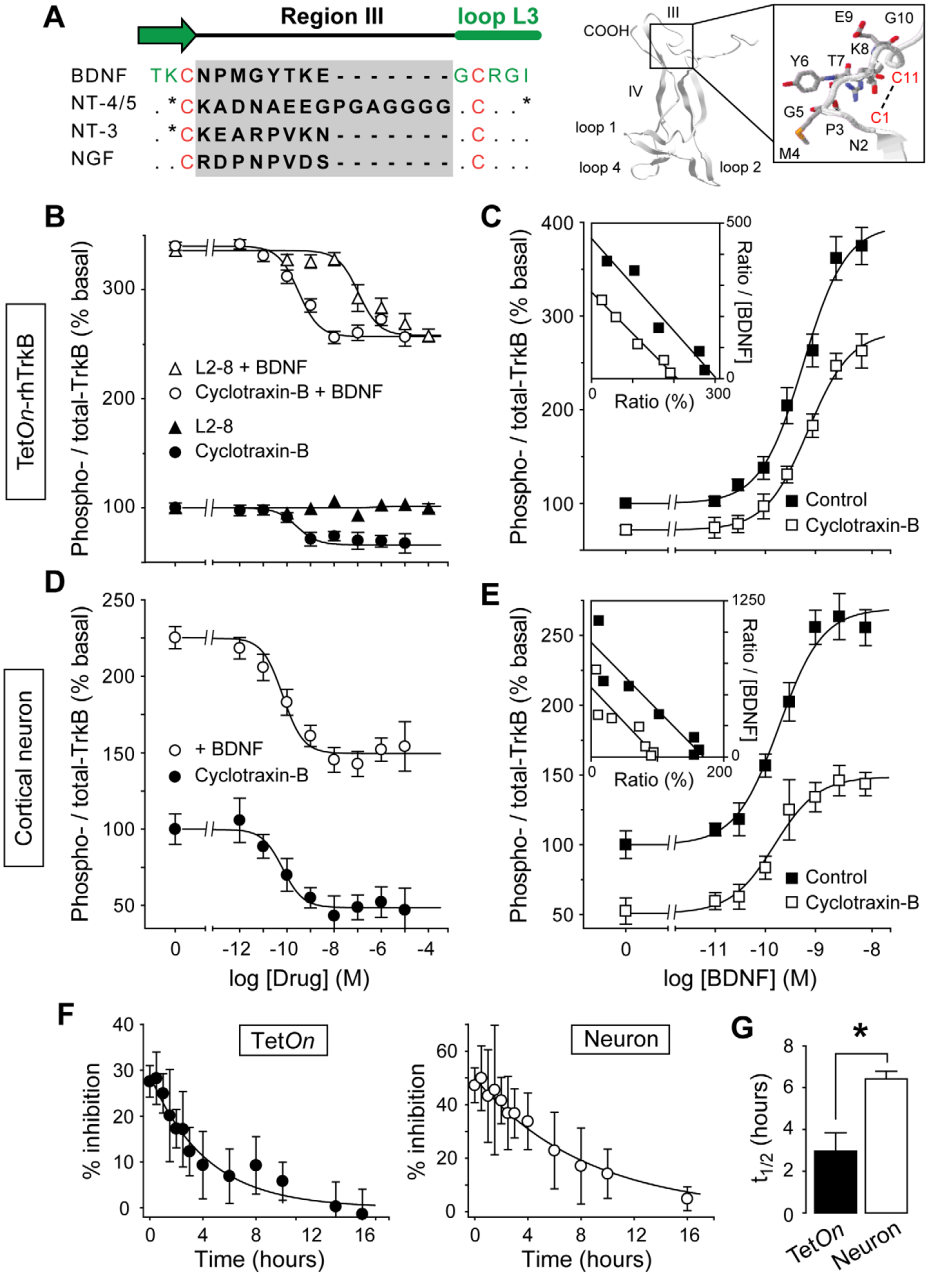
Cyclotraxin-B is a highly potent allosteric inhibitor of TrkB receptor with long-lasting effects. (**A**) Design of cyclotraxin-B. (*Left*) Sequence alignment of the four neurotrophins on the highly variable region III (gray box). The two cysteine residues used for cyclization are in red. *Point*, conserved residues; *asterisk*, highly similar residues. (*Right*) 3-D structure of a BDNF monomer (adapted from PDB entry *1bnd*) and sequence of cyclotraxin-B. Variable regions are indicated and higher magnification of region III is boxed. The position of the disulfide bond is shown as a dashed line. (**B-E**) Characterization of TrkB inhibition by cyclotraxin-B using KIRA-ELISA assays in Tet*On*-rhTrkB cells (**B,C**) and in cortical neurons (**D,E**). (**B,D**) Increasing concentrations of cyclotraxin-B (**B**, *n* = 6; **D**, *n* = 5) or L2-8 (*n* = 3) were added to the cells with or without BDNF. (**C,E**) BDNF concentration-response experiments with or without cyclotraxin-B (**C**, 1 µM, *n* = 6; **E**, 100 nM, *n* = 6). Addition of cyclotraxin-B resulted in a significant uncompetitive antagonism (**C**, F_1,285_ = 88.0, *P*<0.0001; **E**, F_1,279_ = 199.9, *P*<0.0001) and did not change BDNF EC_50_ (**C**, BDNF 672±92 pM, + cyclotraxin-B 749±91 pM; **E**, BDNF 186±50 pM, + cyclotraxin-B 178±52 pM), as shown by Eadie-Hofstee plotting of the data (**insets**). Results are expressed as the ratio between phospho- and total-TrkB in percentage of basal value. Data are mean ± s.e.m. (triplicates, *n* = 6), except for **insets** where data are mean. (**F**) Slow reversibility of cyclotraxin-B inhibition in Tet*On*-rhTrkB cells and in cultured neurons. After 30-min exposure to cyclotraxin-B, cells were rapidly washed and incubated in KIRA-ELISA medium for increasing times before the addition of BDNF. Data are mean ± s.e.m. (triplicates, *n* = 4) and are expressed in percentage of inhibition. (**G**) Means ± s.e.m. of half times obtained in (**F**). * *P* = 0.02.

To design small modulators of the TrkB receptor, we used a strategy that rapidly produces and isolates mimetic peptides by direct proteolysis of mature BDNF. Among several proteases, endoproteinase Glu-C V8 appeared best suited at producing sequences of interest distributed along the BDNF sequence (**Fig. 1** **and** **Table 1**). Proteolytic fragments were purified and identified using a HPLC system connected to a mass spectrometer. Since the main purpose of this study was to develop modulators active on human TrkB receptor, fragments were then assessed in presence of BDNF on the human TrkB receptor, which was expressed in CHO cells in an inducible-manner (Tet*On*-rhTrkB). For that purpose, we developed a modified version of the previously described KIRA-ELISA [28], a rapid, sensitive and high-capacity assay that quantifies TrkB activation in ELISA microtiter plates by measuring phosphorylation of its tyrosine residues [see **Data S1** and **Fig. S1,S2**]. Four fractions were found to significantly decrease BDNF-induced TrkB activity (**Fig. 1C**), including Fragment #(05), corresponding to region III. This fragment was of major interest compared to others because of its very low molecular weight (∼1,200 Da) and highly specific sequence in BDNF. We therefore used both sequence and structure of region III as a basis for the design of cyclotraxin-B, which has been cyclized via terminal cysteine residues to mimic the native structure of this region in BDNF (See **Fig. 2A**).

**Table 1:** 

Fragment	Corresponding region	m/z [M+H]+ (+Cys-CAM)^a^	Position	Peptide sequence
**#(01)**	N-Terminus	1024.6	1–9	**HSDPARRGE**
		(-)		
**#(02)**	-	952.04	10–18	LSVCDSISE
		(1009.45)		
**#(03)**	Loop L1	2279.59	19–40	WV**TAADKKTAVDMSGGT**VTVLE
		(-)		
**#(04)**	Loop L2	1814.11	41–55	**KVPVSKGQL**KQYFYE
		(-)		
**#(05)**	Region III	1271.47	56–66	TKC**NPMGYTKE**
		(1328.59)		
**#(06)**	Loop L4	6220.31	67–119	GCRGIDKRHWNSQCRTTQSYVR
	+ C-Terminus	(6485.32)		ALTM**DSKKRIG**WRFIRIDTSCVC**TLTIKRGR**

a All cysteine residues have been treated with iodoacetamide to form carbamidomethyl-cysteine (Cys-CAM)

### Cyclotraxin-B binds TrkB and alters both basal and BDNF-induced activity with high potency

**Figure 3:**
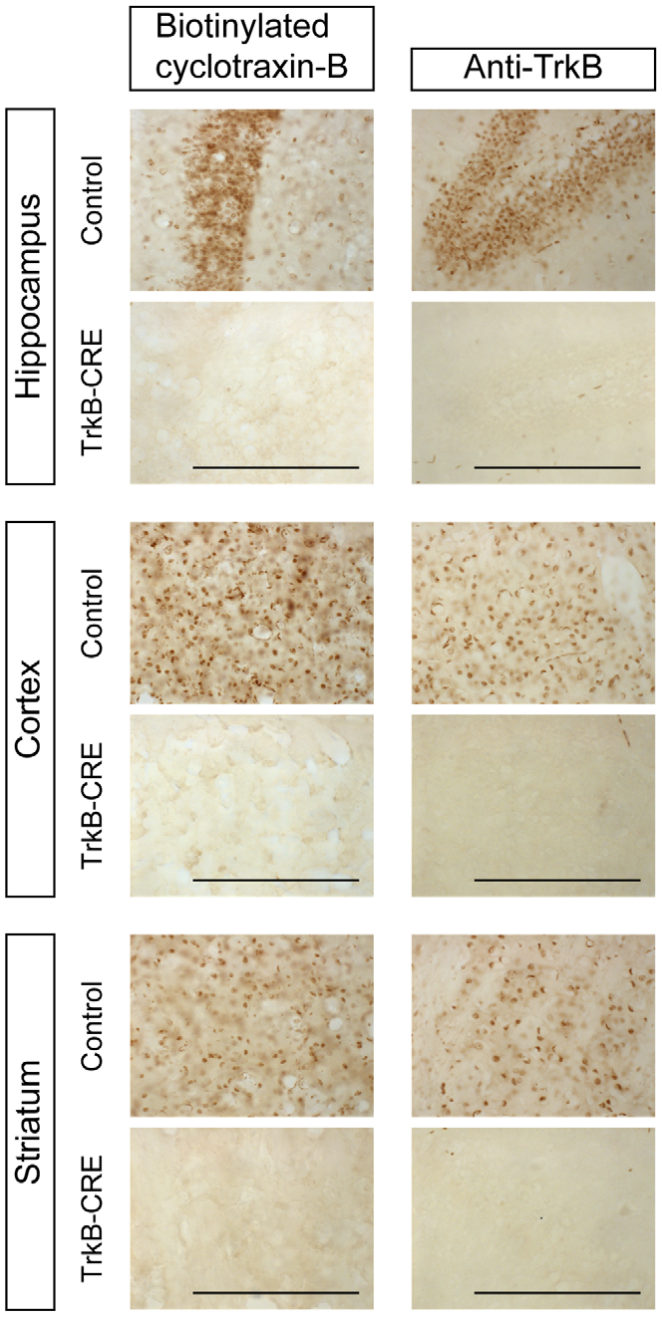
Cyclotraxin-B interacts with TrkB. Fixed slices from adult control and transgenic CamKIIa-CRE x TrkB flox/flox (TrkB-CRE) mice were incubated overnight with biotinylated cyclotraxin-B or anti-TrkB antibody. Regions of the forebrain in which the expression of TrkB is knocked out in the transgenic mice are shown (*Left to right*: dentate gyrus of hippocampus, prefrontal cortex, dorsal striatum; 40× magnification). Data presented for biotinylated cyclotraxin-B are those obtained with 0.1 µM of but are similar with concentrations up to 1 µM. Scale bar, 200 µm.

In a first set of experiments, cyclotraxin-B was assayed on human TrkB receptors using the recombinant Tet*On*-rhTrkB system. Cyclotraxin-B was found to inhibit BDNF-induced TrkB activity through a non-competitive mechanism with a high potency (IC_50_ = 0.30±0.07 nM; **Fig. 2B,D**). When compared to peptide L2-8, a BDNF-mimetic peptide previously described by others [22], cyclotraxin-B proved to have a potency three-order of magnitude higher, in KIRA-ELISA (L2-8 IC_50_ = 108±63 nM; **Fig. 2B,D**). Binding of [^125^I]-BDNF was not altered by cyclotraxin-B (**Fig. S3**), suggesting further that cyclotraxin-B is an allosteric modulator of the receptor by acting through TrkB binding sites that are not critical for the interaction with BDNF but rather involved in its activation capacity. This hypothesis was further confirmed by binding studies using biotinylated cyclotraxin-B. In fact, to test whether cyclotraxin-B is able to interact with TrkB, we performed histochemical staining using a biotinylated version of the peptide on slices issued from either control or transgenic mice lacking the TrkB receptor in the forebrain. This transgenic mouse line was created by breeding CamKIIa-CRE mice [29] to floxed TrkB homozygous mice [30]. The resulting CamKIIa-CRE TrkB flox/flox mice were then crossed to TrkB flox/flox mice to generate 50% CamKIIa-CRE TrkB flox/flox mice (mutants) and 50% TrkB flox/flox mice (control littermates). Because of the selectivity of the expression of CRE recombinase using the CamKIIa promoter, the TrkB gene is only inactivated in the forebrain. As shown in **Figure 3**, whereas cyclotraxin-B is detected in different structures of the forebrain of control mice (*e.g.* dorsal striatum, cortex or hippocampus), no or very weak staining was observed in the same regions of conditional TrkB knockout mice. These observations are comparable to the staining obtained in parallel with a selective TrkB antibody. Together, these results demonstrate that cyclotraxin-B selectively interacts with TrkB without altering the binding of BDNF.

The pharmacological properties of cyclotraxin-B were then tested in native conditions, using primary cultures of mouse cortical neurons. Contrary to Tet*On*-rhTrkB cells, cortical neurons naturally express TrkB receptors and its co-receptor, p75^NTR^. p75^NTR^ is known to alter TrkB conformation and provide greater discrimination for its cognate ligand, BDNF [1], as illustrated by the difference in both affinity and activity observed between BDNF and NT-3 in neurons but not in Tet*On*-rhTrkB cells (**Figs. S2 and S3**). Accordingly, when cyclotraxin-B was tested on native TrkB receptors in cortical neurons, both amplitude of inhibition and potency were enhanced with a 1.6-fold greater inhibition (−56.7±8.8% in neurons *vs.* −34.8±6.4% in *TetOn*-rhTrkB cells) and a one-order of magnitude higher potency than that observed in Tet*On*-rhTrkB cells (IC_50_ = 67.1±18.9 pM), while still remaining non-competitive (**Fig. 2C,E** **and Fig. S3**). Analysis of the reversibility of the inhibitory effects elicited by cyclotraxin-B also demonstrated kinetics differences between Tet*On*-rhTrkB cells and neurons. The t_½_ of cyclotraxin-B was ∼3 hours in Tet*On*-rhTrkB cells and ∼6 hours in neurons (**Fig. 2F,G**), both demonstrating long-lasting effects of cyclotraxin-B after its withdrawal. These relative high values may reflect the high affinity of cyclotraxin-B for recombinant and native TrkB. Similarly, the significant difference in t_½_ may be explained by the difference in potencies observed between the two cell types.

**Figure 4:**
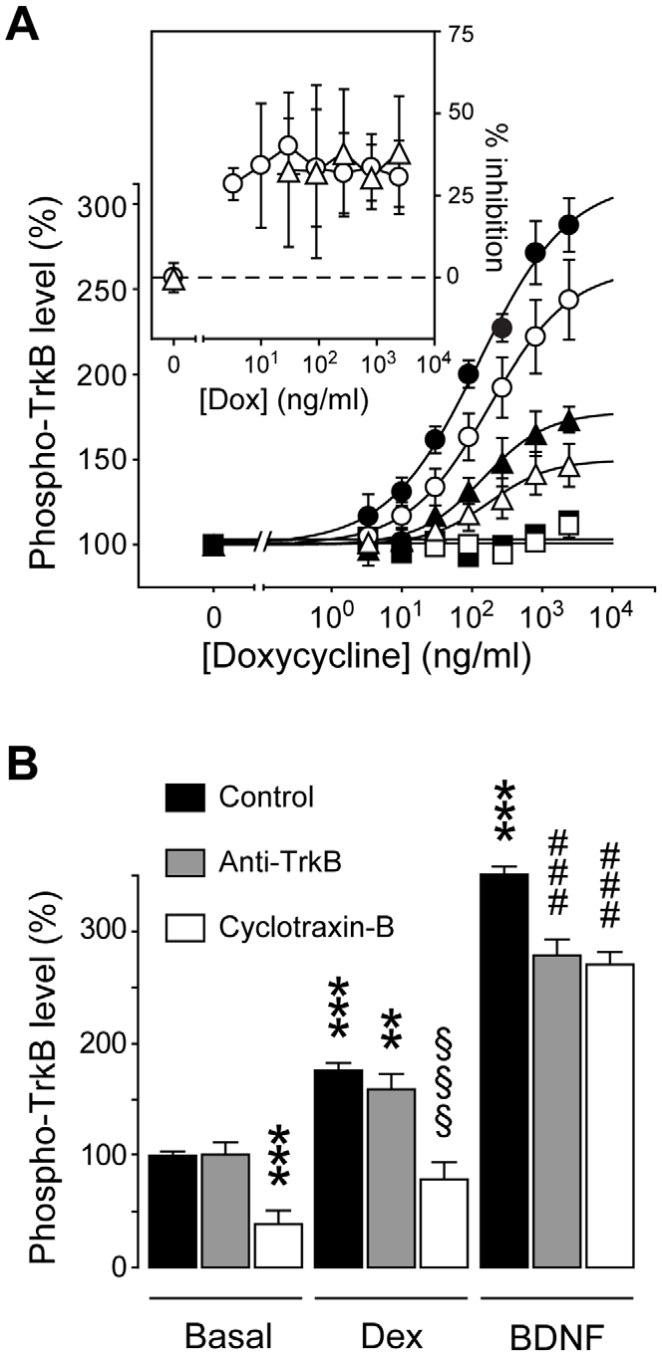
Cyclotraxin-B inhibits both BDNF-dependent and -independent TrkB activity. (**A**) Cyclotraxin-B inhibition of density-related TrkB activity. Cells were induced with doxycycline (see **Fig. S4B**) and were treated with BDNF (4 nM), cyclotraxin-B (1 µM) and K252a (1 µM) before KIRA-ELISA analysis (• BDNF, ○ BDNF+cyclotraxin-B, ▴ basal, Δ cyclotraxin-B, ▪ K252a, □ K252a+cyclotraxin-B). **Inset** shows amplitude of inhibition of BDNF-induced and basal activities. Data are mean ± s.e.m. (triplicates, *n* = 3). Results are expressed in percentage of values obtained in non-induced cells. (**B**) Cyclotraxin-B inhibition of glucocorticoid-dependent TrkB activity in neurons. Cortical neurons were treated with control medium, dexamethasone (1 µM, 2 h) or BDNF (4 nM), in presence or not of cyclotraxin-B (1 µM) or a monoclonal anti-TrkB antibody (30 µg/ml). Data are mean ± s.e.m. (triplicates, *n* = 6) and are expressed in percentage of basal values; ** *P*<0.01, *** *P*<0.001, compared to basal/control condition; §§§ *P*<0.001, compared to dexamethasone alone; ### *P*<0.001, compared to BDNF condition.

Contrary to L2-8, cyclotraxin-B also decreased a BDNF-independent (basal) TrkB activity in both recombinant cells and neurons with the same relative inhibition as in presence of BDNF (Tet*On*-rhTrkB cells, −32.5±8.0% and IC_50_ = 0.28±0.08 nM; neurons, −53.0±5.8% and IC_50_ = 65.7±21.7 pM; **Fig. 2B-E**). Endogenously synthesized BDNF was not involved in this apparent basal activity since neutralizing anti-BDNF antibody did not alter cyclotraxin-B effects (**Fig. S4A**) and no BDNF was detectable by ELISA. This spontaneous activity may result from transactivations by G-protein coupled receptors (GPCR) [2], [3], zinc [5], endocannabinoids [6] but also from increases in receptor density that lead to spontaneous autophosphorylation [31]. This basal activation of TrkB may selectively occur in densely packed area throughout soma, dendrites and axons, at the plasma membrane but also in intracellular vesicles [32], [33]. Taking advantage of the inducible heterologous Tet*On*-rhTrkB system, we investigated the effect of cyclotraxin-B at different TrkB densities. As expected, a threshold in TrkB expression was needed to detect a basal TrkB activity (**Fig. S4B**) and the relative inhibition of cyclotraxin-B was stable at all TrkB densities, either in presence or in absence of BDNF (**Fig. 4A**). In cortical neurons, it has been shown that glucorticoids such as dexamethasone can indirectly activate TrkB receptors [7]. As shown in **Figure 4B**, dexamethasone significantly triggered TrkB phosphorylation in our cortical neurons cultures. This activation via glucorticoids signaling pathways was fully prevented by cyclotraxin-B, contrary to a functional anti-TrkB antibody capable of inhibiting BDNF-dependent TrkB activity only. These results suggest that not only cyclotraxin-B blocks the effects of BDNF on TrkB but also any direct or indirect processes capable of activating TrkB.

### Cyclotraxin-B alters TrkB-related physiological and pathophysiological cellular events

**Figure 5:**
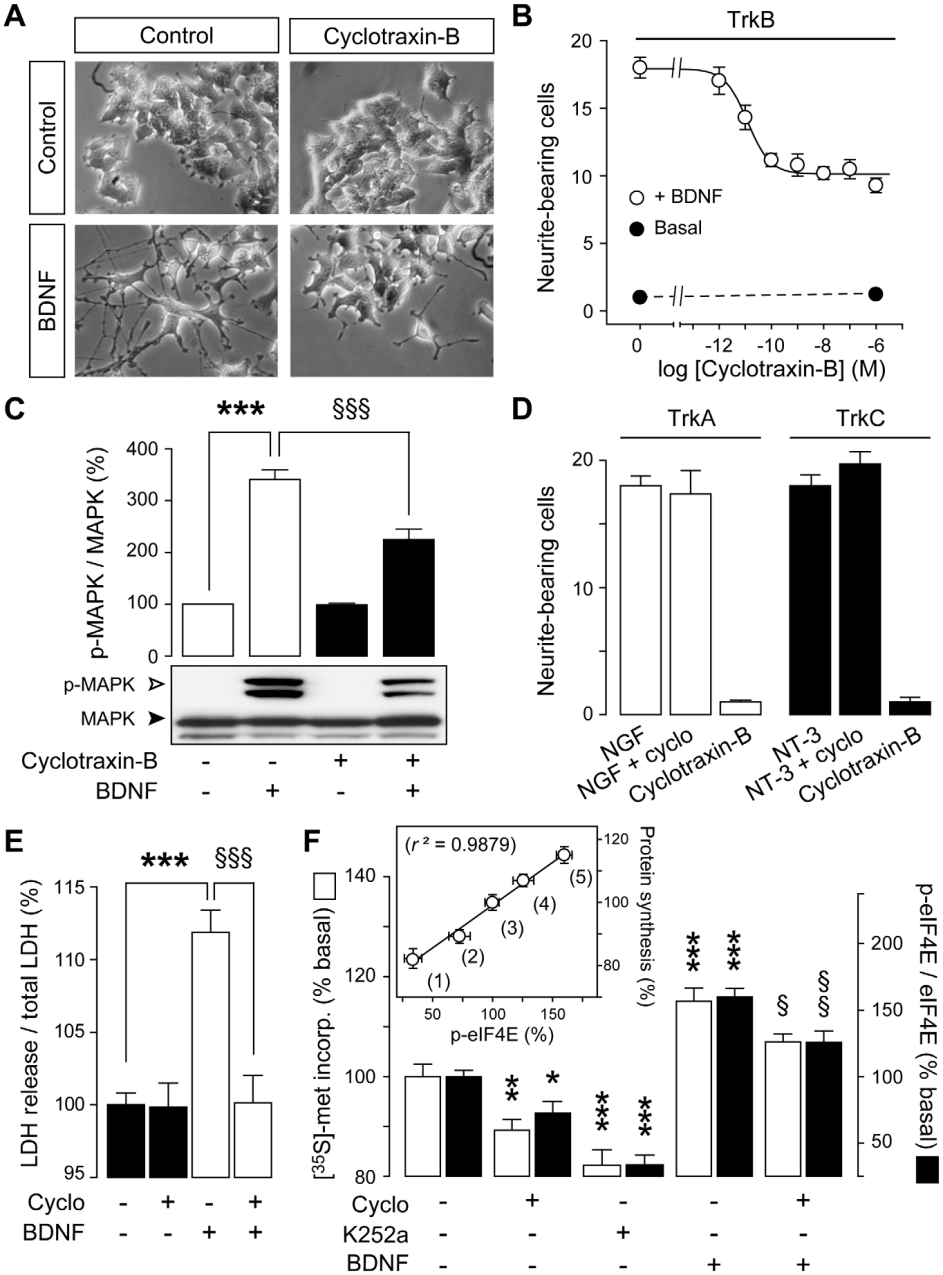
Cyclotraxin-B inhibits normal and deleterious cellular signaling events associated with TrkB but not TrkA nor TrkC. (**A-D**) Cyclotraxin-B inhibits TrkB- but not TrkA- nor TrkC-dependent neurite outgrowth. (**A**) Representative photomicrographs of nnr5 PC12-TrkB cells treated for 48 h with cyclotraxin-B and/or BDNF. 20× magnification. (**B**) Quantitative analysis of BDNF-induced neurite outgrowth in presence of increasing concentrations of cyclotraxin-B. Data are mean ± s.e.m. (sixplicates; *n* = 5). (**C**) Quantitative analysis and representative western blot of total and phospho-MAPK in nnr5 PC12-TrkB cells treated as indicated. Data are mean ± s.e.m. (*n* = 4) and are expressed in percentage of basal condition; *** *P*<0.001 compared to control; §§§ *P*<0.001, compared to BDNF. (**D**) nnr5 PC12-TrkA and -TrkC cells were incubated with cyclotraxin-B (1 µM) in presence or not of NGF or NT-3, respectively. (**E**) Cyclotraxin-B prevents BDNF-induced neurons death. Cortical neurons were treated with cyclotraxin-B or BDNF, as described in methods. Data are mean ± s.e.m. (sixplicates, *n* = 5) expressed in percentage of control. *** *P*<0.001 compared to control; §§§ *P*<0.001, compared to BDNF. (**F**) Cyclotraxin-B inhibits cap-dependent protein translation in cortical neurons. [^35^S]-methionine incorporation into proteins (white bars) was measured in cortical neurons exposed to cyclotraxin-B, K252a and BDNF, as indicated. Phosphorylation of eIF4E was quantified by immunoblots (black bars). Data are mean ± s.e.m. (triplicates, *n* = 5) and are expressed in percentage of basal condition; * *P*<0.05, ** *P*<0.01, *** *P*<0.001, compared to respective basal condition; § *P*<0.05, §§ *P*<0.01, compared to respective BDNF condition. **Inset** shows correlations between TrkB-dependent protein synthesis and eIF4E phosphorylation. (1) K252a, (2) cyclotraxin-B, (3) basal, (4) BDNF + cyclotraxin-B, (5) BDNF.

We next extended the evaluation of the functions of cyclotraxin-B to TrkB-regulated cellular processes. BDNF is known to promote neurite outgrowth, notably in nnr5 PC12-TrkB cells, through MAP-Kinase (MAPK) signaling pathway activation. Addition of BDNF to TrkB/p75^NTR^ co-expressing nnr5 PC12-TrkB cells resulted in increases in neurite length and number of branch points (**Fig. 5A**). The BDNF-induced neurite outgrowth was dose-dependently prevented by cyclotraxin-B with similar pharmacological properties to that of cortical neurons in KIRA-ELISA assays (−49.3±0.5%; IC_50_ = 12.2±8.5 pM) (**Fig. 5A,B**). Application of cyclotraxin-B to nnr5 PC12-TrkB cells dramatically decreased the BDNF-induced phosphorylation of MAPK, proportionally to its effect on neurite outgrowth (**Fig. 5C**). Note that neither spontaneous neurite outgrowth nor basal phospho-MAPK levels could be detected in absence of BDNF, so that no cyclotraxin-B effect could be assessed in this experimental condition. Cyclotraxin-B was selective to TrkB receptors since concentrations up to 1 µM did not affect NGF- nor NT-3-induced neurite outgrowth in nnr5 PC12-TrkA and -TrkC, respectively (**Fig. 5D**). Moreover, morphological analysis ruled out toxicity effects of cyclotraxin-B and no cell death was observed even 72 hours after exposure to 10 µM cyclotraxin-B.

Reports have proposed that a sustained activation of TrkB receptors by BDNF or through GPCR transactivations could lead to enhanced neuronal vulnerability to toxic insults [10], [34], [35], [36], [37]. Although the underlying mechanisms are poorly known, studies performed on cultured cortical neurons have shown that excess BDNF can induce neuronal death through activation of TrkB but not p75^NTR^ [34], [37]. In this context, we tested the effect of cyclotraxin-B in a model of BDNF-induced neuronal necrosis. While repeated exposure to high concentrations of BDNF induced cortical neurons necrosis, treatment with cyclotraxin-B fully prevented BDNF-induced cell death without altering normal neuron survival (**Fig. 5E**). These results demonstrate the neuroprotective effects of cyclotraxin-B in this experimental paradigm. Similar conclusions were drawn from cell survival experiments using MTT (methylthiazolyldiphenyl-tetrazolium bromide) method (**Fig. S5**).

**Figure 6:**
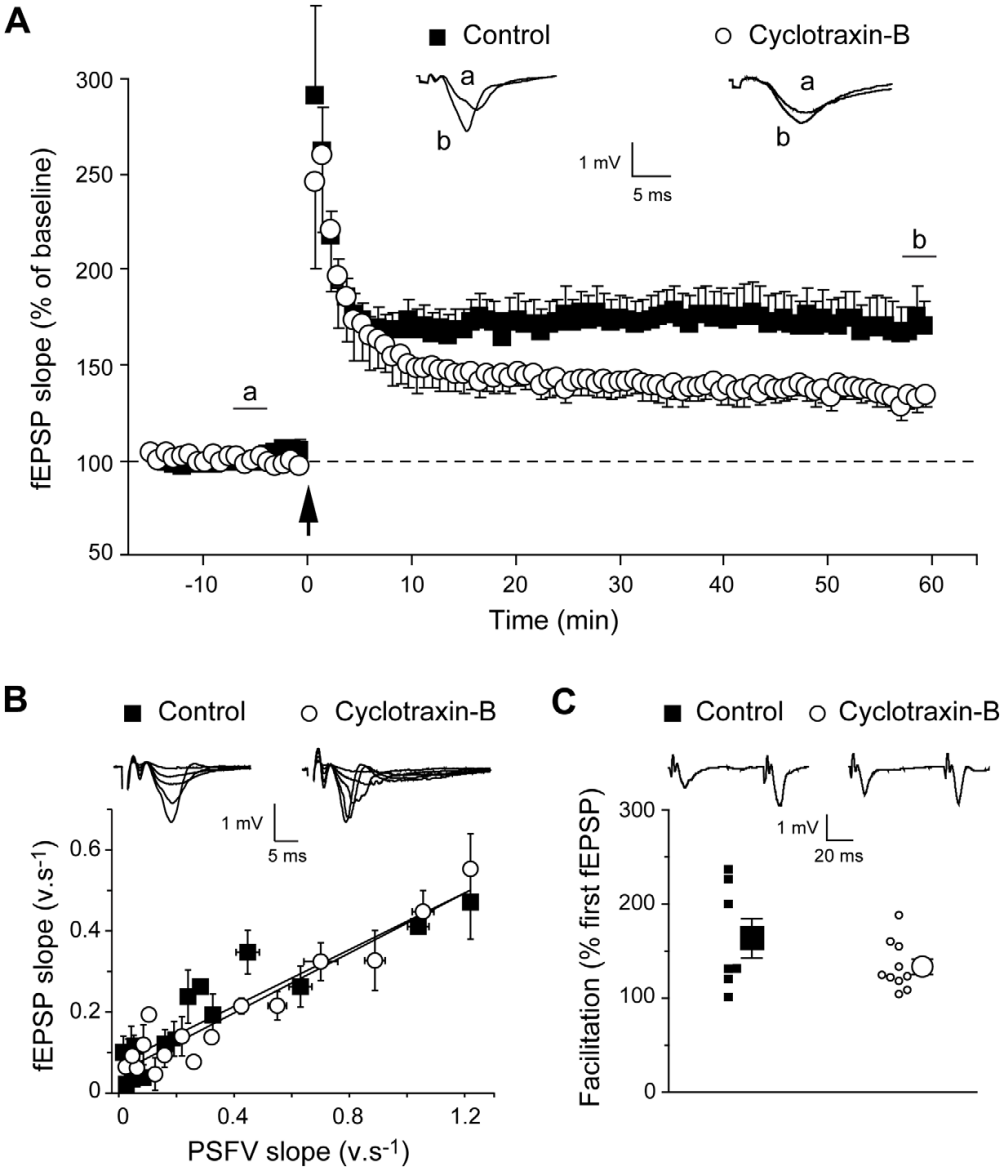
Cyclotraxin-B impairs HFS-induced LTP without affecting basal synaptic transmission. (**A**) Effect of prior exposure to cyclotraxin-B on tetanus-induced LTP in the CA1 area. Grouped recordings are shown before and after tetanic stimulation (arrow) for control and cyclotraxin-B-treated hippocampal slices. Representative fEPSPs recorded 5 min before (a) and 60 min after (b) LTP induction are shown. Results are expressed in percentage of baseline response and are the mean ± s.e.m. (*n* = 8−9 slices). (**B**) Input-output curves plotting of fEPSP slopes against presynaptic fiber volley (PSFV) slope in control (*r* ^2^ = 0.84; *n* = 7) and cyclotraxin-B-treated slices (*r* ^2^ = 0.92; *n* = 10). Individual traces obtained for control and cyclotraxin-B-treated slices are shown. (**C**) Scatter plot depicting the facilitation ratio obtained in control (*n* = 7) and cyclotraxin-B-treated slices (*n* = 10). Mean ± s.e.m. is indicated by the large symbol with the error bars while smaller symbols represent data obtained in each experiment. Representative sweeps obtained with control and cyclotraxin-B-treated slices are shown.

BDNF and TrkB also play a central role in the long-term modulation of synaptic plasticity [8]. We thus tested cyclotraxin-B on BDNF-induced protein synthesis in neurons, a process that is linked to induction and maintenance of the late events of long-term potentiation (LTP). As shown in **Figure 5F**, the BDNF-induced increase in newly synthesized protein level was significantly reduced by cyclotraxin-B. Of interest is the decrease in basal rate of protein synthesis by cyclotraxin-B, which reproduced half of the inhibition induced by the non-selective Trk inhibitor K252a. This suggests that a part of basal rate of protein synthesis depends on basal TrkB activity in neurons. Neither BDNF nor cyclotraxin-B showed any effect on non-induced Tet*On*-rhTrkB cells (**data not shown**). The phosphorylation of the eukaryotic initiation factor 4E (eIF4E) is known to underlie the TrkB-dependent protein synthesis that occurs during synaptic modulation by BDNF, as shown in **Figure 5F** **(see also Fig. S6)**. Cyclotraxin-B significantly reduced the level of phosphorylated eIF4E protein both in the absence and in the presence of the neurotrophin. Remarkably, these effects were linearly correlated to those observed on protein synthesis, thus suggesting a direct link between these two cellular events and TrkB modulation. Since LTP at the Schaffer collateral-CA1 synapses is known to require BDNF derived from presynaptic CA3 neurons [29], attempts were made to determine whether cyclotraxin-B was able to affect tetanus-induced LTP in this neural network. As shown in **Figure 6A**, while HFS applied to hippocampal slices induced LTP in both control and cyclotraxin-B-treated slices (169±15%, *P*<0.001, and 134±6%, *P*<0.01, respectively; 50–60 min after HFS), this potentiation was significantly decreased in presence of cyclotraxin-B (*P*<0.01, 50–60 min after HFS). As previously observed by Kang and colleagues using function-blocking TrkB antiserum [38], cyclotraxin-B did not alter either synaptic strength or presynaptic functions (**Fig. 6B,C**). These results indicate that cyclotraxin-B counteracts TrkB-dependent LTP without affecting normal synaptic transmission at Schaffer collateral-CA1 synapses. Altogether, these results demonstrate that cyclotraxin-B alters signaling pathways associated to both BDNF-induced and basal TrkB activities, in normal and/or deleterious conditions.

### Systemic injections of cyclotraxin-B to mice result in TrkB inhibition in the brain and alters anxiety-related behavior

**Figure 7:**
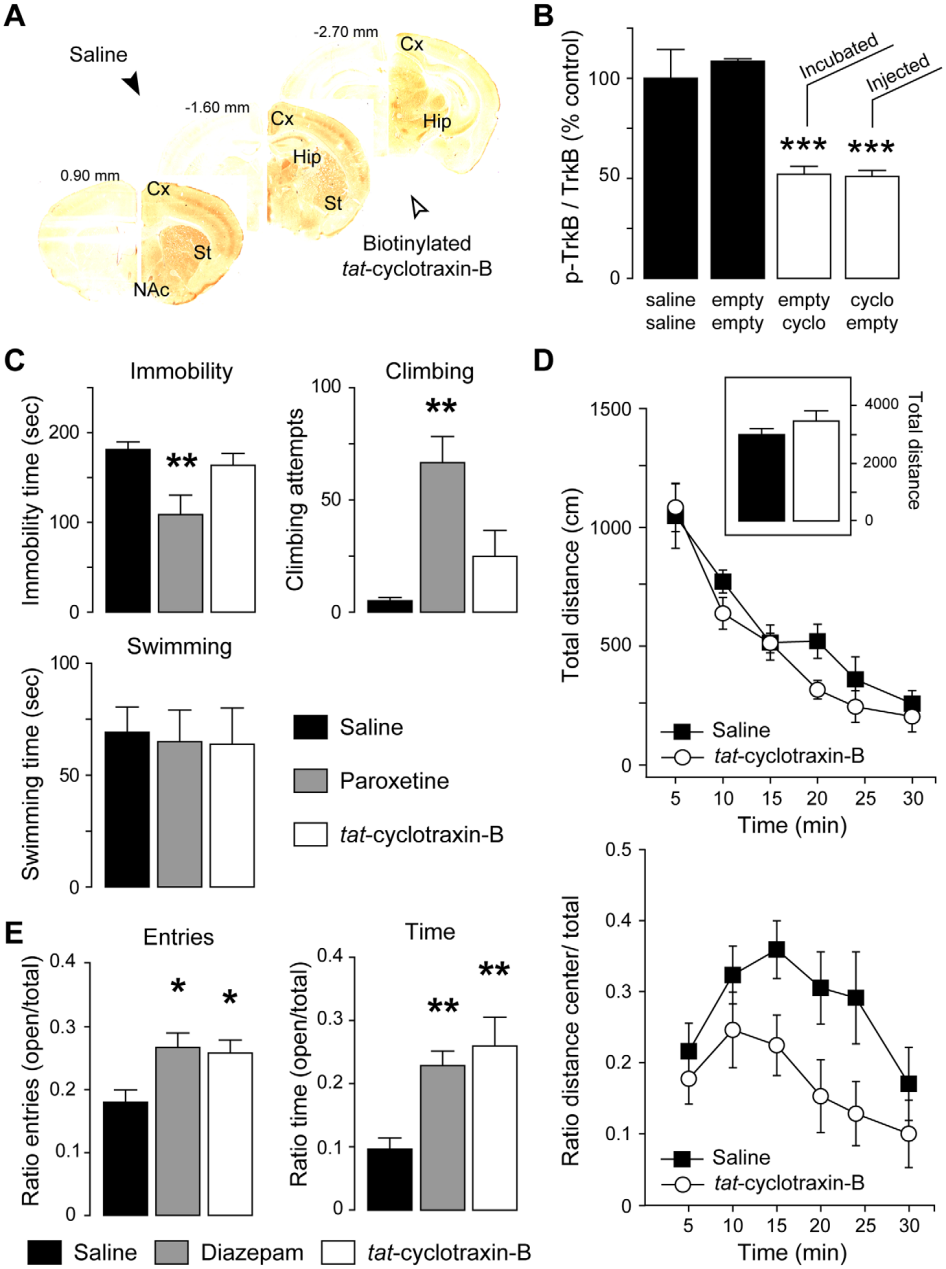
Cyclotraxin-B is active *in vivo* after systemic injections and demonstrates anxiolytic-like but not antidepressant-like effects. (**A**) Brain localization of biotinylated *tat*-cyclotraxin-B after intravenous injections. Cx, cortex; St, striatum; NAc, Nucleus Accumbens; Hip, hippocampus. (**B**) KIRA-ELISA analysis of brain TrkB receptors from mice treated with saline (sal), *tat*-empty (empty) or *tat*-cyclotraxin-B (cyclo) as described in **Figure S8**. Results are expressed in percentage of control condition. Data are mean ± s.e.m. (sixplicates, *n* = 18 from 6 mice). *** *P*<0.001, compared to saline. (**C**) No antidepressant-like action of *tat*-cyclotraxin-B. Mice treated with saline solution (*n* = 11), *tat*-cyclotraxin-B (*n* = 6) or paroxetine (*n* = 8) were tested for anti-depressive behaviors using FST. Results are mean ± s.e.m. ** *P*<0.01, compared to saline. (**D**) Anxiolytic-like effect of *tat*-cyclotraxin-B with no effect on locomotor activity in the open field. Mice treated with either saline (*n = 10*) or *tat*-cyclotraxin-B (*n = 10*) were subjected to an open field. (*Up*) The total distance traveled in the periphery and in the center was measured during 30 min. (**Inset**) Cumulative distance measured during the 30-min trial does not show any locomotor alterations. (*Down*) The normalized distance traveled in the bright center is expressed as a ratio between the distance traveled in the center and the total distance. For the normalized distance in center, there was a significant effect of treatment (F_1,108_ = 0.337, *P*<0.001) and time (F_5,108_ = 0.068, *P* = 0.01). Results are mean ± s.e.m. (**E**) Anxiolytic-like effect of *tat*-cyclotraxin-B comparable to that of diazepam. Mice that received saline solution (*n* = 7), *tat*-cyclotraxin-B (*n* = 10) or diazepam (*n* = 7) were assessed for anxiety-like behaviors in the EPM procedure. Data are expressed as a ratio between entries in open arms over total number of entries and ratio between time in center over total time. Results are mean ± s.e.m. * *P*<0.05, ** *P*<0.01, compared to saline.

The overall purpose of the present study was to develop a TrkB modulator that can be administered by systemic injection and then to assess the resulting behavioral central effects. In order to allow its delivery to the brain after intravenous injections, we fused the non-toxic transduction domain of the *tat* protein from the HIV type 1 [39] to cyclotraxin-B (*tat*-cyclotraxin-B). KIRA-ELISA showed that the fusion with *tat* did not alter the pharmacological properties of cyclotraxin-B (**Fig. S7**). In contrast, the fusion with *tat* even enhanced the efficacy of cyclotraxin-B to inhibit TrkB in brain slices (cyclotraxin-B, −29.8±5.6%, *n* = 9; *tat*-cyclotraxin-B, −51.2±3.6%, *n* = 12; *P*<0.05), while *tat*-empty (a *tat* peptide lacking the cyclotraxin-B sequence) did not produce any effect. This enhancement may reflect the plasma membrane permeability of the *tat*-fused cyclotraxin-B that facilitates its penetration into the slice and/or targeting of intracellular active TrkB receptors. We then treated adult mice with *tat*-cyclotraxin-B or *tat*-empty following a double-injection procedure (see protocol in **Fig. S8**). Four hours after the last intravenous injection, *tat*-cyclotraxin-B was detected in many brain structures that normally express TrkB (cortex, hippocampus, striatum, nucleus accumbens; **Fig 7A**), demonstrating the stability of the compound *in vivo*. More interestingly, the total level of TrkB phosphorylation in *tat*-cyclotraxin-B-treated mice was dramatically decreased as compared to saline or *tat*-empty-treated animals (**Fig. 7B**). Similar results were obtained amongst all these brain regions (**not illustrated**). Western blot analysis using phospho(Y816)-TrkB antibody showed comparable effects with a reduction of 37.7±4.7% in band intensity (**Fig. S8B**), suggesting that cyclotraxin-B inhibits both PLCγ and Shc binding sites. Remarkably, even with very different administration kinetics (minutes for incubation; hours for injection), the decrease in TrkB activity was not significantly different when *tat*-cyclotraxin-B was injected to animals or directly added to the slice (injection, 50.9±3.1%; slice incubation, 52.0±3.9%; **Fig. 7B**). This latter observation thus illustrates the long-lasting effects of *tat*-cyclotraxin-B *in vivo*.

BDNF and TrkB have been shown to regulate development of depression-like phenotypes and anxiety-related behaviors [14], [40], [41], [42], depicting TrkB as a promising target for the development of new therapeutic compounds [13], [43]. In this context, we focused the study of adult mice injected with *tat*-cyclotraxin-B on depression-like and anxiety-like behaviors. First, mice were subjected to the forced swim test (FST), a test that is commonly used for its predictive validity regarding antidepressant compounds. In this test, *tat*-cyclotraxin-B was compared to paroxetine, a well-characterized antidepressant compound. As for many antidepressant compounds, mice acutely treated with paroxetine demonstrated significant decreased time of immobility and increased climbing attempts (**Fig. 7C**). This escape behavior was not observed for mice that received saline solution or *tat*-cyclotraxin-B, suggesting that cyclotraxin-B does not possess any antidepressant-like properties when injected acutely. Noteworthy, swimming capabilities were not affected in mice treated with *tat*-cyclotraxin-B, suggesting no locomotor alterations. In a second set of experiments, *tat*-cyclotraxin-B was assessed for its potential anxiolytic-like properties. Treated mice were tested in the open field and in the elevated-plus maze (EPM), two tests used for their predictive validity regarding anxiolytic compounds. In the open field, mice injected with *tat*-cyclotraxin-B did not show any alterations in locomotor activity, confirming our previous observations (**Fig. 7D**). Moreover, *tat*-cyclotraxin-B-injected mice spent significantly more time in the center compartment compared to saline controls, suggesting anxiolytic effects of the compound. To confirm this putative anxiolytic property, effects of acute treatment with *tat*-cyclotraxin-B were then assessed using EPM and compared to diazepam, a benzodiazepine commonly used to treat anxiety in humans. Remarkably, mice that received *tat*-cyclotraxin-B exhibited an anxiolytic profile similar to that of diazepam. In fact, both compounds significantly increased the number of entries and the time spent in open arms with comparable amplitude of effects (**Fig. 7E**). Overall, these behavioral studies further demonstrated the specificity of central effects elicited by *tat*-cyclotraxin-B after intravenous injections and confirmed its pharmacological potential *in vivo*, for its use in animal models of brain disorders.

## Discussion

Since the discovery of the first antidepressant and antipsychotic medications fifty years ago, few novel therapeutic agents have been developed for the treatment of psychiatric diseases. Most of the time, new treatments are based on improving existing treatment strategies rather than targeting new molecular pathways. There is an increasing literature that proposes TrkB receptor signaling as a promising target for the treatment of psychiatric disorders [13], [14], [15], [16], but also epilepsy [9], neurodegenerative diseases [10], [11], [44], and even cancer [45]. In consequence, as the first potent and specific modulator of TrkB receptors that can be easily administered *in vivo*, cyclotraxin-B opens new avenues for therapeutic interventions.

The proposed involvement of BDNF or TrkB in diseases is mostly based on an excess or a lack of the BDNF/TrkB coupling that was measured either in human post-mortem tissue or in genetic animal models. The conclusions drawn from these data are not always coherent, especially for those regarding psychiatric diseases, such as schizophrenic psychoses for instance [16]. Therefore, it will be difficult to predict the effect of a modulator of TrkB activity in such cases. Cyclotraxin-B represents a great tool to evaluate the consequences of systemic pharmacological interventions on TrkB receptors in pathologies with altered BDNF/TrkB signaling. An example of such consequences is shown here: global targeting of brain TrkB receptors by intravenous injections of cyclotraxin-B has effects on anxiety- but not depression-related behaviors. Our observations are in line with two recent studies demonstrating that mice susceptible to stress-related disorders after chronic social defeat have an overactivity of BDNF/TrkB signaling in their reward system [40], [41]. This suggests that acute systemic interventions using cyclotraxin-B may preferentially act on the reward system in the brain, resulting at the behavioral level in anxiolytic-like activities rather than on the stress axis and its antidepressive effects [13]. Such specificity in the behavioral effects of cyclotraxin-B may be explained by its capacity to alter the basal activity of TrkB receptors. Brain regions that possess a high basal activation level will thus be more sensitive to cyclotraxin-B. Our results are also consistent with previous studies using transgenic mice lacking TrkB receptors for which no alteration in depressive-like behaviors could be observed [46], [47]. In addition, the anxiolytic- but not antidepressant-like profile may reflect the difference in the kinetics of treatment for both conditions, with acute effects for anxiolytic compounds versus chronic effects for antidepressant molecules. Overall, this study gives a first insight to the predictive pharmacological class of cyclotraxin-B, which appears to behave as an anxiolytic drug similar to other benzodiazepines rather than as an antidepressant compound.

The sustained effect elicited by cyclotraxin-B after transient application both *in vitro* and *in vivo* is a result of the high affinity and slow reversibility of the interaction with the receptor. It may also be due to its small size that reduces protease-sensitivity. However, long-term treatments with TrkB modulators may have unexpected consequences on receptor internalization and recycling. In fact, such modulation may alter the plasma membrane-bound TrkB receptor level, making it difficult to predict the long-term effects of cyclotraxin-B. As well, cyclotraxin-B proved to behave differentially on TrkB if p75^NTR^ is co-expressed or not. Numerous reports demonstrated that the functional cooperation between p75^NTR^ and Trk receptors is responsible for a better discrimination in binding for a specific neurotrophin and higher amplitude of response. In a same way, in presence of p75^NTR^, cyclotraxin-B, like BDNF, gains in binding affinity, amplitude of inhibition and kinetics of action. It has been proposed that coexpression of p75^NTR^ and Trk receptors may induce receptors aggregation in lipid rafts, convergence of signaling pathways or recruitments of different intracellular adaptors [48], [49]. Since cells in the central nervous system express TrkB alone or together with p75^NTR^, one can expect differential amplitude of effects of cyclotraxin-B depending on the targeted region. As a partial allosteric inhibitor, cyclotraxin-B should avoid the deleterious effects of a full inhibitor. Indeed, BDNF and its receptor have important trophic and protective effects on neuronal cells so that a minimal level of TrkB activity must be preserved, which is warranted if cyclotraxin-B is to be used therapeutically. Moreover, it is noteworthy that under some circumstances BDNF promotes cell death. For instance, reports have demonstrated that necrosis of cultured cortical neurons can be induced by BDNF, a process abolished by inhibiting TrkB with antisense oligonucleotides or K252a [34] and absent in cultures made from cortices of TrkB-null mice [35]. Therefore, in conditions in which hyperactivity of the BDNF/TrkB system increases the vulnerability of neurons to excitotoxic insults [10], [34], [35], [36], cyclotraxin-B can be used as a neuroprotective agent by maintaining the receptors in a minimal active state.

**Figure 8:**
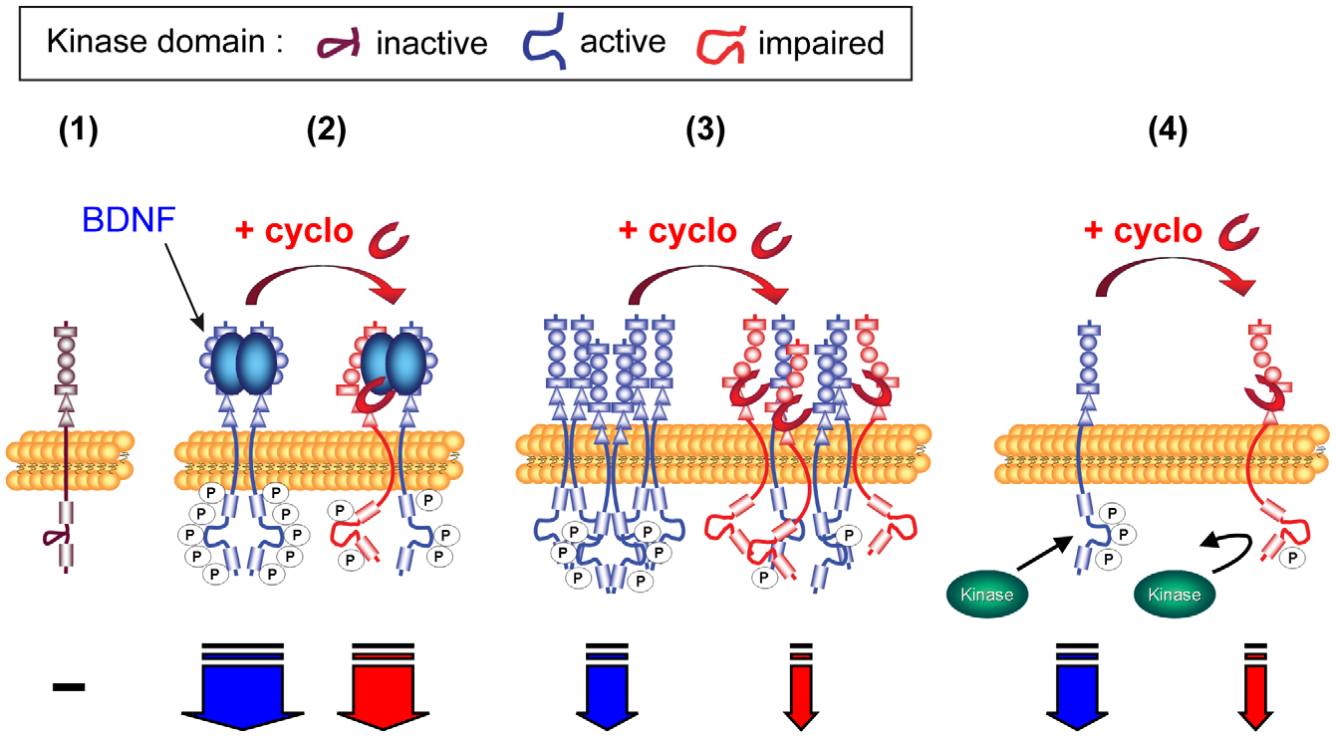
Proposed mechanism of cyclotraxin-B inhibition depending on TrkB activation state. (**1**) Inactive receptor monomer, (**2**) BDNF-induced activation, (**3**) High density-induced activation. (**4**) Src kinase-mediated transactivation. Cyclotraxin-B binds to TrkB sites different from those of BDNF and induces a less-active transconformation state of the receptor, as schematized by the shape of activation loops and the number of phosphorylated residues (circled P, global phosphorylation level) on the intracellular kinase domain. This results in a decrease in activation of TrkB downstream signaling cascades, as schematized by the size of descending arrows.

Pharmacological studies performed with KIRA-ELISA demonstrate that cyclotraxin-B not only blocks the effects of BDNF on TrkB but also any direct or indirect processes capable of activating TrkB (*e.g.* spontaneous dimerization or glucocorticoids, respectively). Therefore, cyclotraxin-B may allosterically interfere with TrkB optimal conformation, thereby preventing its proper activation, independently of the mechanism of activation (**Fig. 8**). This specific property makes cyclotraxin-B a great tool for investigating TrkB transactivation mechanisms as well as spontaneous TrkB dimerizations in physiology and pathology. TrkB transactivation processes have been linked to crucial physiological events such as synaptic plasticity [5], neuron migration and morphogenesis [6] and neuroprotection [7]. In addition, basal more than BDNF-dependent TrkB activity may play a critical role in the etiology of some pathologies such as epileptogenesis [9], amyotrophic lateral sclerosis [10] and cancer metastases [50]. An example of the importance of the basal TrkB activity in physiology as revealed by cyclotraxin-B is reported in **Figure 6**. Surprisingly, the effect on the LTP reduction appeared high (from about 170 to 135%) relative to the partial inhibition of cyclotraxin-B. In comparison, Korte *et al.* have reported a reduction within the same range, from about 190% to 135%, using BDNF knockout mice [51]. This high effect could be due to the fact that TrkB inhibition is more general since cyclotraxin-B inhibits not only the effect of BDNF on TrkB but also the basal TrkB activation. These results suggest an important component of the BDNF-independent activity of TrkB receptors in the regulation of synaptic plasticity, as proposed by others [5]. Finally, cyclotraxin-B inhibits the two main signaling pathways downstream TrkB with similar amplitude of effects. In fact, activation of TrkB triggers the phosphorylation of two Tyrosine residues that form binding sites for Shc (Y515) and PLCγ (Y816). KIRA-ELISA assays performed with a pan-phospho-tyrosine antibody first demonstrated that cyclotraxin-B inhibits the overall phosphorylation level of TrkB. Then, we showed that cyclotraxin-B inhibits the activation of the MAPK pathway as well as subsequent neurite outgrowth, two signaling downstream the binding of protein Shc to phosphorylated Y515. Finally, western blots performed with a specific phospho(Y816)-TrkB antibody demonstrated that cyclotraxin-B also inhibits the activation of the PLCγ binding site and subsequent processes such as the modulation of LTP. Therefore, this study shows that cyclotraxin-B is able to inhibit the two active sites of TrkB (p-Y816/PLCγ and p-Y515/Shc) as well as downstream processes associated to these pathways (Shc/MAPK/neurite outgrowth and PLCγ/synaptic plasticity).

Altogether, these observations suggest that cyclotraxin-B represents a promising tool for the study of the BDNF/TrkB signaling in pathological and physiological processes and could serve as a lead compound for the development of novel therapeutic strategies.

## Materials and Methods

### Ethics statement

All experimental procedures were carried out in accordance with the INSERM committee guidelines and European Communities Council Directive (86/609/EEC, 24 November 1986) regarding the care and use of animals.

### Plasmid constructs and cell lines

Plasmid constructs were generated by using standard cloning techniques and were confirmed by sequencing. The human recombinant TrkB receptor was fluorescently tagged at its C-terminus with the Enhanced cyan fluorescent protein (rhTrkB-ECFP) by cloning the full human TrkB sequence [52] (kindly provided by G. M. Brodeur) into the pECFP-N1 expression vector (Clontech). For inducible expression, rhTrkB-ECFP was subcloned into the tetracycline-responsive (Tet*On*) vector pTRE2 (Clontech). Chinese Hamster Ovary cells stably expressing rhTrkB-ECFP (Tet*On*-rhTrkB cells), were produced by transfection of the pTRE2-rhTrkB-ECFP plasmid into CHO-K1 Tet*On* cells (Clontech) followed by clone selection in 0.25 mg/ml hygromycin B (Invitrogen) and 0.1 mg/ml geneticin (G418; Invitrogen). Resistant cells were selected based on both fluorescence intensity and KIRA-ELISA profile after an overnight incubation in presence of 1000 ng/ml doxycycline (Clontech). Cortical neurons were prepared and cultured from E16 mouse embryos as described previously [53]. The nnr5 PC12-TrkA, nnr5 PC12-TrkB and nnr5 PC12-TrkC cells (kindly provided by M. V. Chao) are NGF non-responding mutant PC12 cells stably transfected with TrkA, TrkB and TrkC cDNA, respectively [54].

### Enzymatic cleavage of BDNF and purification/characterization of fragments

Ten micrograms (0.37 nmoles) of mature recombinant human BDNF (Peprotech) were dissolved in 400 mM ammonium bicarbonate buffer (pH 8.0) and cysteine bridges were reduced by adding 10 mM DTT for 15 min at 50°C and alkylated by 15 mM iodoacetamide for 15 min at room temperature. Digestion buffer was diluted to 100 mM ammonium bicarbonate buffer before adding endoproteinase Glu-C (type V8; Sigma) in a 1∶100 (enzyme:protein) ratio for 24 h at 37°C. Digestion mixture was dried, reconstituted in 50 µl water and divided in two samples. The first sample (10 µl, ∼74 pmole) was used for the separation and identification of fragments using Liquid Chromatography/ElectroSpray Ionization-Mass Spectrometry (LC/ESI-MS). Mass spectra were acquired on a LCQ Advantage ion trap mass spectrometer (Thermo Electron). On-line HPLC separation of the fragments was accomplished with an Atlantis column (2.1×150 mm, Waters) packed with dC18 adsorbent (5 µM, 300 Å) (mobile phase A, 0.1% formic acid; mobile phase B, 80% acetonitrile, 0.1% formic acid). The first 2.5 min at 0% mobile phase B were derivatized to waste. The following non-linear gradient was then used: 0–15% B over 4.5 min, 15–30% B over 23 min, 30–50% B over 10 min, 50–100% B over 2 min, 100% B over 3 min, 100-0% B over 1 min (flow rate: 250 µl/min). The column was washed within 4 min with 100% solvent B before reequilibration with mobile phase A. Digestion fragments were sequentially fragmented into the ion trap by collision-induced dissociation using an isolation width of 2 and a relative collision energy of 35%. Mass spectrometry and mass spectrometry fragmentation data allowed an unambiguous identification of the different fragments using predictive m/z and the Bioworks software. The second sample (∼30 µl) was used for separation and isolation of fragments into aliquots. Using exactly the same HPLC parameters than previously, we saw to it that one fragment was distributed in only one fraction and reciprocally, that one fraction contained only one fragment at a time. Fractions were dried, washed two times, reconstituted in 50 µl water and stored at -20°C. Fractions (∼1 µM final) were then tested in competition with BDNF (20 ng/ml) using KIRA-ELISA. Four distinct BDNF digestions reproducing the same LC/ESI-MS profile were performed. Each fraction was tested in triplicate in six different KIRA-ELISA assays.

### Peptides design and synthesis

See SI for methods for the production of mimetic peptides. Cyclotraxin-B, L2-8, *tat*-cyclotraxin-B and *tat*-empty were purchased from BioS&T. Peptide sequences were as follows: cyclotraxin-B, CNPMGYTKEGC (cyclized by disulfide bridge); L2-8, CVPVSKGQLC [22]; *tat*-cyclotraxin-B, YGRKKRRQRRRCNPMGYTKEGC ; biotinylated *tat*-cyclotraxin-B, Biotin-YGRKKRRQRRRCNPMGYTKEGC ; *tat*-empty, YGRKKRRQRRR. Peptides were purified to the highest grade by reverse-phase HPLC (>98.5%).

### KIRA-ELISA

TrkB activity was quantified using the previously described KIRA-ELISA assay [28] with slight modifications. Cortical neurons were seeded on polyornithin-coated flat-bottom 96-well culture plates (12×10^4^ cells per well) and cultured eight days at 37°C in 5% CO_2_. TetOn-rhTrkB cells were seeded on flat-bottom 96-well culture plates (4×10^4^ cells per well) and incubated overnight with 1000 ng/ml doxycycline (**Figs. S1,S2**) except when indicated. Fluorescence levels in TetOn-rhTrkB cells were verified before each assay. Both cell types were carefully washed four times with Dulbecco's modified Eagle's medium (DMEM, Invitrogen) before being pre-treated for 30 min with ligands and stimulated for 20 min with 4 nM BDNF, except when indicated in the figures, in DMEM containing 0.5% BSA and 25 mM Hepes (pH 7.4). Assay was stopped by removing the medium on ice and membranes were solubilized using KIRA-ELISA solubilization buffer (NaCl 150 mM, Hepes 50 mM, Triton X-100 0.5%, thimerosal 0.01%, sodium orthovanadate 2 mM, supplemented with a cocktail of protease inhibitors) for 1 h at room temperature. Solubilized membranes were transferred for 2 h at room temperature onto ELISA microtiter plate (Nunc Maxisorp) pre-coated overnight at 4°C with appropriate antibodies [polyclonal anti-GFP (1∶5000, Clontech) for TetOn-rhTrkB, polyclonal anti-TrkB (1 µg/ml, Upstate) for cultured neurons and brain slices, for phosphorylation assays; monoclonal anti-GFP (1∶3000, Chemicon) for TetOn-rhTrkB, monoclonal anti-TrkB (1∶1000, BD Biosciences) for cultured neurons and brain slices, for total TrkB quantification]. Tyrosine phosphorylation was revealed by a 2 h incubation with biotinylated anti-phosphotyrosine (4G10, 0.5 µg/ml; Upstate) and a streptavidin-peroxydase system (1∶4000; Amersham Biosciences). Total TrkB was revealed by a 2 h incubation with polyclonal anti-GFP (1∶5000, Clontech) for TetOn-rhTrkB, or polyclonal anti-TrkB (1 µg/ml, Upstate) for cultured neurons and brain slices, followed by appropriate secondary antibodies and a peroxydase system. Absorbance was read at 450 nm after addition of 3,3′,5′,5-tetramethyl-benzidine substrate (Sigma) and acidification with 1N hydrochloric acid. Background signal was defined as indicated in **Fig. S2**.

### BDNF quantification

Endogenous BDNF was quantified in cortical neuron cultures by using the BDNF Emax immunoassay system (Promega).

### Fluorescence

ECFP fluorescence quantification was read at 485 nm using a Mithras LB 940 counter (Berthold Technologies).

### [^125^I]-BDNF binding studies

Two micrograms of recombinant human BDNF (Peprotech) were dissolved in 50 mM phosphate buffer (pH 7.0). Iodination was allowed by incubation with 0.5 mCi [^125^I] and chloramine T and reaction was stopped by addition of sodium disulfite. The labeled BDNF was purified by PD-10 desalting columns (Amersham Biosciences) and fractions containing the [^125^I]-BDNF were pooled (63.3 µCi/µg). Competition binding studies were done mainly as previously described [55]. Briefly, 2.5−3×10^5^ cells or neurons were carefully washed with ice-cold binding buffer (DMEM containing 0.5% BSA and 25 mM Hepes) before being treated as indicated in the figures. Cells were then washed four times with ice-cold binding buffer and lyzed with 1N NaOH. Radioactivity was counted in a 1260 MultiGammaII counter (LKB Wallac).

### Immunoblot analysis

Cell lysates were prepared in boiling SDS (2%, w/v) as described previously [56] and subjected to SDS-PAGE. Detection and analysis of eIF4E, MAPK and Akt phosphorylation were done using anti-total and -phospho-eIF4E antibodies (1∶1000, Cell Signaling), anti-total and -phospho-MAPK antibodies (1∶1000, Cell Signaling) or anti-total and–phospho (Ser473) Akt antibodies (1∶1000, Santa Cruz; 1∶1000, Cell Signaling, respectively), respectively. For TrkB expression analysis, cortical neurons were cultured for eight days and Tet*On*-rhTrkB cells were incubated overnight with doxycycline. Cells were solubilized as for KIRA-ELISA assays and lysates were centrifuged at 14,000×g for 20 min at 4°C. Cleared supernatant was collected and proteins were subjected to SDS-PAGE. Detection of rhTrkB-ECFP and neuronal mouse TrkB were performed using anti-GFP polyclonal antibody (1∶5000, Clontech) and anti-rodent TrkB polyclonal antibody (1 µg/ml, Upstate), respectively. TrkB phosphorylation was quantified using anti-phospho-TrkB antibody (kindly provided by M.V. Chao) [57]. Proteins were visualized using appropriate HRP-conjugated secondary antibodies and films were quantified by densitometry.

### Immunofluorescence

Cortical neurons seeded on polyornithin-coated cover slips were fixed in 4% paraformaldehyde for 20 min and washed in glycine buffer (0.1 M, pH 7.4). Detection was performed using anti-rodent TrkB polyclonal antibody (1 µg/ml, Upstate) or anti-rodent MAP-2 monoclonal antibody (1∶500, Millipore) in Saponine buffer (0.05%) supplemented with 0.2% BSA and 10% heat-inactivated fetal bovine serum and revealed using Alexa488-conjugated (1∶100, Interchim) or Cy3-conjugated (1∶200, Jackson) appropriate secondary antibodies.

### Immunohistochemistry

Mice were injected twice with 200 µg of biotinylated *tat*-cyclotraxin-B. Three hours after the first injection, animals were deeply anesthetized with pentobarbital and perfused intracardiacally with a solution of saline followed by 4% paraformaldehyde. Brains were postfixed for 1 h in 4% paraformaldehyde and cut in 40-µm sections with a vibratome. Sections obtained at the same levels as for *in vivo* KIRA-ELISA analysis (see **Fig. S8**) were rinsed in PBS and incubated in TBS containing 0.05% Tween-20 with VectaStain ABC Elite kit (Vector Laboratories). Signal was revealed using 3,3′ diaminobenzidine (DAB). Non-injected mice were used as negative controls for endogenous biotin.

### Histochemistry using biotinylated cyclotraxin-B

Control and CamKIIa-Cre TrkB fl/fl adult mice (see below) >12 weeks-old were transcardially perfused with 4% paraformaldehyde. Brains were post-fixed overnight in 4% PFA, cryopreserved in 30% sucrose and cryosectioned into 40 µm sagittal sections. Sections were stored in PBS with azide. Biotinylated cyclotraxin-B was incubated at 0.1 or 1 µM overnight at 4°C. Sections were rinsed in PBS and signal was revealed using VectaStain ABC Elite kit (Vector Laboratories). Signal was revealed using 3,3′ diaminobenzidine (DAB). In parallel, adjacent sections were blocked in normal mouse serum and incubated with monoclonal mouse anti-TrkB (1∶250; BD Bioscience) overnight at 4°C. Signal was revealed as for biotinylated cyclotraxin-B. In both conditions (biotinylated cyclotraxin-B and anti-TrkB), a slight staining could be sometimes observed for some slices (only at 1 µM for cyclotraxin-B). This signal is consistent with other studies using the similar transgenic mice [30] and may be attributed to TrkB-expressing glial cells and/or a not total knockout.

### Neurite outgrowth assessment

nnr5 PC12 cells were prepared as described elsewhere [58]. nnr5 PC12-TrkB, -TrkA and -TrkC cells were treated with cyclotraxin-B (100 nM) and BDNF (1 nM), NGF (2 nM) or NT-3 (10 nM), respectively. The number of cells bearing neurites longer than 2 cells in diameter was microscopically determined in each counting field (two fields per well, three wells per condition). Counting was performed blind each 24 h for three days. Data presented in the figures are those obtained for 48 h but are similar at 24 h and 72 h.

### Metabolic labeling with [^35^S]-methionine

Measurement of [^35^S]-methionine incorporation was mainly performed as described previously [56] Cortical neurons were washed twice and incubated with DMEM containing 0.5% BSA and 25 mM Hepes for 5–6 h. Neurons were incubated for 20 min with 4 nM BDNF in the presence of 4 µCi/ml of [^35^S]-methionine (1000 Ci/mmol, Amersham Biosciences). Cyclotraxin-B (100 nM) and K252a (10 µM) were added for 30 min prior to BDNF (4 nM) stimulation. Neurons were washed twice and protein were precipitated with trichloroacetic acid (TCA; 10%, w/v). Amino acid uptake into neurons and incorporation into proteins were estimated by counting the radioactivity in the supernatant and the pellet, respectively. [^35^S]-Methionine incorporation was calculated as the ratio of TCA-precipitable to TCA-soluble radioactivity. None of the treatments altered [^35^S]-methionine uptake into neurons.

### Assessment of cell injury

Neurotoxicity experiments were performed on cortical neurons (25×10^4^ cells per well in 24-well plates) cultured in presence of glial cells in Neurobasal medium supplemented with B27 (Gibco) for 10–14 days. Mature cultures were then washed carefully and serum-free medium containing cyclotraxin-B (200 nM) or BDNF (4 nM), alone or in combination, was added to the cells each 24 h for 72 h. Neuronal necrosis was determined by measuring the leakage of LDH with respect to total LDH using the kit of Sigma, as described by the manufacturer. Necrosis was estimated as a function of LDH released compared to total LDH. Cell survival was quantified by measuring the reduction of MTT into a blue formazan precipitate that is subsequently solubilized in dimethyl sulphoxide and read at 560 nm.

### Animals

Adult male Sprague-Dawley rats and Swiss mice (2–3 months-old and 4–6 weeks, respectively; Janvier) were used. Forebrain-specific TrkB transgenic mice (CamKIIa-Cre TrkB fl/fl mice) used for biotinylated cyclotraxin-B histochemistry have been created by breeding CamKIIa-Cre mice [29] to floxed TrkB homozygotes mice [30] to generate CamKIIa-Cre+ TrkB fl/wt mice and then crossed to TrkB fl/fl mice to generate CamKIIa-Cre TrkB fl/fl mice. The resulting line had low neonatal survival but surviving animals matured unremarkably. Animals were maintained on a controlled light-dark cycle at a constant temperature (22±2°C) with *ad libitum* access to food and water.

### Electrophysiological recordings

Hippocampal slices (400 µm) were prepared as previously described [59]. Cyclotraxin-B was directly diluted in medium (1 µM) and slices were incubated for at least 30 min. Slices were then transferred to a submersion-type recording chamber equilibrated with 95% O_2_, 5% CO_2_, and submerged in a stream of medium containing 1 µM cyclotraxin-B for ∼20 min prior to I/O curves and PPF recordings or to LTP induction (see below). Extracellular recordings were performed at room temperature in apical dendritic layers of CA1 area using glass micropipettes filled with 2 M NaCl with a resistance of 2–6 MΩ. Field excitatory postsynaptic potentials (fEPSPs) were evoked by electrical stimulation of CA1 afferent Schaffer collaterals and commissural fibers in the stratum radiatum. Test stimuli (100 µs duration) were adjusted to get 30% of maximum fEPSP slope and applied every 15 seconds.

#### Long-term potentiation (LTP)

LTP was induced by applying high frequency stimulation (HFS) to Schaffer collaterals (two 1-sec trains at 100 Hz separated by 20 seconds) after baseline recording (at least 15 minutes or until stable). The magnitude of fEPSPs was determined by measuring the slope of fEPSPs. Three successive fEPSPs were averaged (each fEPSP was normalized to the mean of fEPSPs recorded before tetanus) and plotted across time using Pclamp9 software (DIPSI). Recording after single pulse testing was performed for at least 60 minutes following tetanus.

#### Input/Output (I/O)

Curves were constructed to assess the responsiveness of AMPA/Kainate glutamate receptor subtypes-dependent responses to electrical stimulation in slices. Slopes of three averaged presynaptic fibre volleys (PFVs) and fEPSPs were plotted as a function of stimulation intensity (400–2200 µA).

#### Paired-pulse facilitation (PPF)

PPF of synaptic transmission induced by paired-pulse stimulation was monitored at 40 ms inter-stimulus intervals. PPF was quantified by normalizing the slope of the second fEPSP by the slope of the first one.

### Intravenous delivery of cyclotraxin-B and *in vivo* KIRA-ELISA analysis

Adult Swiss mice (20–25 g) received a double 90-min-interval i.v. injection of saline buffer, *tat*-cyclotraxin-B (2×200 µg) or *tat*-empty (2×200 µg). Three hours after the first injection, mice were decapitated, brains quickly removed on ice, 300-µm coronal sections were prepared with a vibratome at three levels (bregma 0.90 mm: caudate putamen/nucleus accumbens level; bregma −1.60 mm: dorsal hippocampus level; bregma −2.70 mm: ventral hippocampus level, see **SI** **Fig. 8A**) and placed in a holding chamber for one hour. Slices were then incubated in Ringer's solution supplemented with *tat*-cyclotraxin-B or *tat*-empty (1 µM) in holding chamber saturated with 95% O_2_, 5% CO_2_. After 45 min incubation, BDNF (4 nM) was added for 20 min. Experiments were stopped by quick removing of the medium on ice. Slices were homogenized in KIRA-ELISA solubilization buffer (NaCl 150 mM, Hepes 50 mM, Triton X-100 0.5%, thimerosal 0.01%, sodium orthovanadate 2 mM, supplemented with a cocktail of protease inhibitors) and membranes were left solubilized overnight at 4°C. Protein concentrations were determined and equal amounts of proteins were loaded in each well for KIRA-ELISA assays (see above). The following antibodies combination was used for phosphorylation assays: TrkB capture with polyclonal anti-TrkB (1 µg/ml); tyrosine phosphorylation quantification with biotinylated anti-phosphotyrosine (4G10, 0.5 µg/ml) and a streptavidin-peroxydase system (1∶4000). The following combination was used for total TrkB quantification: TrkB capture with monoclonal anti-TrkB (1∶1000); total TrkB quantification with polyclonal anti-TrkB (1 µg/ml). Absorbance at 450 nm was read after addition of TMB and acidification.

### Behavioral testing

Mice were treated as described above before being subjected to behavioral studies. Predictive estimation for anxiolytic properties of *tat*-cyclotraxin-B was assessed using open field and elevated plus-maze (EPM). Antidepressant-like action of *tat*-cyclotraxin-B was assessed with the forced-swim test (FST). Effects of acute treatment with *tat*-cyclotraxin-B on spontaneous locomotion, exploration and reactivity to a novel open field was assessed in plexiglas activity chambers (Med Associates; 45×45×31 cm). Mice were placed into the center of the open field, and activity was recorded for 30 min. Testing took place under bright ambient light conditions to increase the anxiety component of the center areas of the field (defined as the central 15×15 cm portion). Mice subjected to EPM received a double 90-interval i.v. injection of *tat*-cyclotraxin-B or saline buffer before being placed on the platform of the EPM and assessed for 5 min (26×5×36 cm). The diazepam control group consisted of saline buffer double-treated mice that further received a 30-min i.p. injection of diazepam (1 mg/kg). Mice were assessed for number of entries and amount of time spent in open versus closed arms. The FST procedure was applied to treated mice as described by Porsolt *et al*. (see ref. [60]). Mice were dropped individually into glass cylinder (height, 25 cm; diameter, 10 cm) containing 10 cm water height, maintained at 23–25°C. Animals were tested for a total of 6 min. The total time of swimming, the number of climbing attempts and the time of immobility was recorded during the last 4 min of the session, after 2 min of habituation. The paroxetine control group consisted of saline buffer double-treated mice that further received a 30-min i.p. injection of paroxetine (2 mg/kg). Behaviors were recorded on videotape and scored blind.

### Statistical analysis

Concentration-response and competition curves were obtained using an iterative least-squares method derived from that of Parker & Waud (1971). Eadie-Hofstee plotting of the data provided estimates for BDNF EC_50_ in presence or not of cyclotraxin-B and IC_50_ values of peptides L2-8, cyclotraxin-B and *tat*-cyclotraxin-B. KIRA-ELISA competition studies, protein synthesis, eIF4E phosphorylation, HFS-induced LTP and survival assessments (MTT and LDH release methods) were analyzed using *one-way* ANOVA followed by Newmann-Keuls multiple comparison *post-hoc* test. Concentration-response curves and Eadie-Hofstee plots were analyzed using *two-way* ANOVA. Significance over time in locomotor activity studies was given using *two-way* ANOVA followed by Bonferroni's *post-hoc* test. Cyclotraxin-B reversibility and 30-min cumulative counts in locomotor activity were analyzed using Student's *t* test. *One-*way ANOVA followed by *post-hoc* Dunnett's test was used to evaluate statistical significance compare to saline group in EPM and FST procedures. *n* represents the number of independent experiments.

## Supporting Information

Data S1 Supplementary information
(0.04 MB DOC)

Figure S1 Cells systems for the analysis of recombinant and neuronal TrkB receptors. (A) Representative fluorescence photomicrographs of Tet*On*-rhTrkB inducible cells following an overnight incubation with (*middle panel*) or without (*left panel*) doxycycline and confocal TrkB immunofluorescence in mouse cortical neurons (*right panel*). Scale bar, 55 µm. (B) Western blot analysis of TrkB expression in Tet*On*-rhTrkB cells treated with increasing concentrations of doxycycline and in cultured mouse cortical neurons. One band was detected ({similar, tilde operator } 170 kDa, closed arrowhead) for recombinant human TrkB fused to ECFP whereas two distinct bands (95 and 145 kDa, open arrowhead) were revealed for neuronal TrkB. Doxycycline dose-dependently induced the expression of rhTrkB-ECFP in Tet*On*-rhTrkB cells and was used at the optimal concentration of 1000 ng/ml in all further experiments. (C) Representative western blot analysis of total and phosphorylated TrkB in TetOn-rhTrkB cells and neurons, and total and phosphorylated MAPK and Akt in cortical neurons, after treatment with or without BDNF (1 nM).
(2.27 MB TIF)

Figure S2 Pharmacology of recombinant and neuronal TrkB receptor using KIRA-ELISA. (A,B) KIRA-ELISA concentration-response curves for BDNF, NT-3 and NGF in Tet*On*-rhTrkB cells and in cultured cortical neurons. Results are expressed as mean ± s.e.m. of raw absorbance read at 450 nm in six independent experiments performed in triplicate. (C,D) Inhibition of TrkB phosphorylation by K252a in both Tet*On*-rhTrkB cells and cortical neurons. Cells were treated for 20 min with K252a prior to treatment with (closed circle) or without (closed square) BDNF (4 nM). K252a was dissolved in dimethyl sulphoxide (DMSO); we verified that DMSO did not affect TrkB activity. As values obtained with 10 µM K252a are not different from those obtained in non-induced Tet*On*-rhTrkB cells (with BDNF, open circle; without BDNF, open square; C), background signal was defined as the value obtained with 10 µM K252a in KIRA-ELISA studies in both cell types. Data are mean ± s.e.m. of four experiments performed in triplicate and results are expressed as a ratio between phospho-TrkB and total TrkB in percentage of basal. (E) Western blot analysis of concentration-response curve for BDNF in Tet*On*-rhTrkB cells. Representative blots are shown for phospho-TrkB, TrkB and β-Tubulin (*left*) as well as quantitative analysis of band intensity (*right*). White and black arrows show the active form of TrkB. Data are mean ± s.e.m. of three experiments and results are expressed as a ratio between phospho-TrkB and total TrkB in percentage of basal.
(1.05 MB TIF)

Figure S3 Cyclotraxin-B does not compete with [^125^I]-BDNF binding to recombinant nor to neuronal TrkB receptors. Effect of unlabeled BDNF (closed circle), NT-3 (closed triangle), NGF (closed square), peptide L2-8 (open triangle) and cyclotraxin-B (open circle) on [^125^I]-BDNF binding to recombinant and neuronal TrkB receptors. Adherent cells were pre-incubated 60 min at 4°C with increasing concentrations of unlabeled BDNF, NT-3, NGF, peptide L2-8 and cyclotraxin-B before an additional 2 hours incubation at 4°C with 200 pM [^125^I]-BDNF. Each point represents the results from two independent experiments performed in triplicate.
(0.25 MB TIF)

Figure S4 Basal activity of TrkB receptors in absence of BDNF. (A) Absence of endogenous BDNF in cultured cortical neurons. Neuronal cells were incubated 24 hours with a neutralizing anti-BDNF antibody before treatment with cyclotraxin-B. KIRA-ELISA analysis revealed no significant difference in cyclotraxin-B inhibition with or without pretreatment with anti-BDNF. *P<0.01 compared to their respective basal condition. Data are mean ± s.e.m. (triplicates, *n* = 3). p.i., preincubation. (B) BDNF-independent TrkB activity depends on TrkB density in Tet*On*-rhTrkB cells. Cells were incubated overnight with increasing concentrations of doxycycline. Total and phospho-TrkB were then evaluated using fluorescence quantification and KIRA-ELISA, respectively.
(0.26 MB TIF)

Figure S5 BDNF-induced neuronal necrosis is prevented by cyclotraxin-B. Cyclotraxin-B prevents BDNF-induced neurons death through a NMDA-independent pathway. Cortical neurons were treated with cyclotraxin-B (200 nM), BDNF (4 nM), NMDA (200 µM) or MK-801 (1 µM), as indicated. Data are mean ± s.e.m. (octuples, *n* = 6) expressed in percentage of control. ***P<0.001 compared to control; *$$$P*<0.001, compared to BDNF; *n.s*, non significant.
(0.30 MB TIF)

Figure S6 Cyclotraxin-B inhibits eIF4E phosphorylation in cortical neurons. Cortical neurons were exposed to cyclotraxin-B, K252a and BDNF, as indicated. Phosphorylation of eIF4E was quantified by immunoblots using anti-phospho and anti-total-eIF4E antibodies. A representative western-blot is shown here.
(0.33 MB TIF)

Figure S7 *Tat*-cyclotraxin-B inhibits both recombinant and neuronal TrkB receptors similarly to cyclotraxin-B. Characterization of TrkB inhibition by *tat*-cyclotraxin-B using KIRA-ELISA assays in Tet*On*-rhTrkB cells and in cortical neurons. Increasing concentrations of *tat*-empty and *tat*-cyclotraxin-B were added to the cells for 30 min prior to treatment with or without 4 nM BDNF for 20 min (*tat*-cyclotraxin-B, closed circle; *tat*-cyclotraxin-B + BDNF, open circle; *tat*-empty, closed triangle; *tat*-empty + BDNF, open triangle). Data are mean ± s.e.m. (triplicates, *n* = 4, Tet*On*-rhTrkB; *n* = 2, cortical neurons).
(0.24 MB TIF)

Figure S8 Protocol for *in vivo* KIRA-ELISA analysis after intravenous injection. (A) Adult mice received two i.v. injections of saline buffer, *tat*-empty or *tat*-cyclotraxin-B (1). Brains were then sliced at three levels (bregma 0.90 mm: caudate putamen/nucleus accumbens level; bregma −1.60 mm: dorsal hippocampus level; bregma −2.70 mm: ventral hippocampus level) and each half-slice was treated with BDNF (4 nM) and either *tat*-empty or *tat*-cyclotraxin-B (1 µM) (2). Slices were then solubilized and subjected to KIRA-ELISA analyzes (3). (B) Western blot analysis of *tat*-cyclotraxin-B effect on TrkB activation *in vivo*. Concentration-response curve for BDNF in Tet*On*-rhTrkB cells. Representative western blots of brain phospho-TrkB, total-TrkB and β-Tubulin from mice injected with either *tat*-empty or *tat*-cyclotraxin-B are shown (*up*). White and black arrows show the active form of TrkB. Bands intensity have been quantified (*down*) Data are mean ± s.e.m. (duplicates, *n* = 6 mice/group) expressed in percentage of control. ***P<0.001 compared to control.
(0.95 MB TIF)
