# Supplementary material for: Cyclotraxin-B, the First Highly Potent and Selective TrkB Inhibitor, Has Anxiolytic Properties in Mice
Source: PLoS One. 2010 Mar 19;5(3):e9777. doi: 10.1371/journal.pone.0009777 (PMC2841647; doi:10.1371/journal.pone.0009777)
Supplement: Data S1 — Supplementary information (0.04 MB DOC) [file pone.0009777.s001.doc]

**Data S1**

**Cyclotraxin-B, the first highly potent and selective TrkB inhibitor, has anxiolytic properties in mice**

Maxime Cazorla^1,4,6^, Anne Jouvenceau^2^, Christiane Rose^1^, Jean-Philippe Guilloux^1,5^, Catherine Pilon^1^, Alex Dranovsky^3^ and Joël Prémont^1^

^1^Neurobiology & Molecular Pharmacology, Centre de Psychiatrie et de Neurosciences, UMR-894 INSERM / Université Paris Descartes, Paris, FRANCE

^2^Pathophysiology of Ageing Brain, Centre de Psychiatrie et de Neurosciences, UMR-894 INSERM / Université Paris Descartes, Paris, FRANCE

^3^Department of Psychiatry, Columbia University, New York State Psychiatric Institute, New York, NY, USA

^4^Present address: Department of Psychiatry, Columbia University, New York, NY, USA

^5^Present address: Serotonin & Neuropharmacology, EA 3544, Université Paris-Sud, Chatenay-Malabry, FRANCE

^6^Corresponding author: Maxime Cazorla, Columbia University, Department of Psychiatry, Physicians & Surgeons Bldg, Room 7-431, 630 West 168th Street, New York, NY 10032, USA

Ph : 1-212-342-3115, Fax : 1-212-305-5594, E-mail : [mc3195@columbia.edu](mailto:mc3195@columbia.edu)

The pharmacology of recombinant human and neuronal mouse TrkB receptors was analyzed using KIRA-ELISA assay (**Figs. S1,S2**). As expected, recombinant TrkB in Tet*On*-rhTrkB cells indistinctively responded to BDNF and NT-3 (ratio signal/background = 3.9 for BDNF and 3.8 for NT-3). In TrkB + p75^NTR^ co-expressing cortical neurons, NT-3 behaved as a partial agonist (ratio signal/background = 2.1 for BDNF and 1.7 for NT-3). Similarly, whereas EC_50_ of BDNF and NT-3 remained almost the same in recombinant Tet*On*-rhTrkB cells (BDNF, 614 ± 123 pM; NT-3, 702 ± 134 nM), neuronal TrkB receptors were significantly more selective to BDNF than to NT-3 (BDNF, 208 ± 80 pM; NT-3, 716 ± 167 nM; *P* = 0.0247). NGF did not produce any signal neither in recombinant nor in neuronal cells. BDNF signal was fully reversed by the non-selective tyrosine kinase inhibitor K252a (**Fig. S2**). Assays using K252a also revealed a basal activity for TrkB both in Tet*On*-rhTrkB and neuronal cells. This BDNF-independent K252a-sensitive TrkB activity is of similar amplitude in both systems. Finally, phospho-TrkB quantification by western blots (a method more commonly used to assess TrkB activation) showed a signal/background ratio comparable to that of KIRA-ELISA (3.96 with western blots, 3.94 with KIRA-ELISA).
