# Supplementary figures and images for: Cyclotraxin-B, the First Highly Potent and Selective TrkB Inhibitor, Has Anxiolytic Properties in Mice
Source: PLoS One. 2010 Mar 19;5(3):e9777. doi: 10.1371/journal.pone.0009777 (PMC2841647; doi:10.1371/journal.pone.0009777)

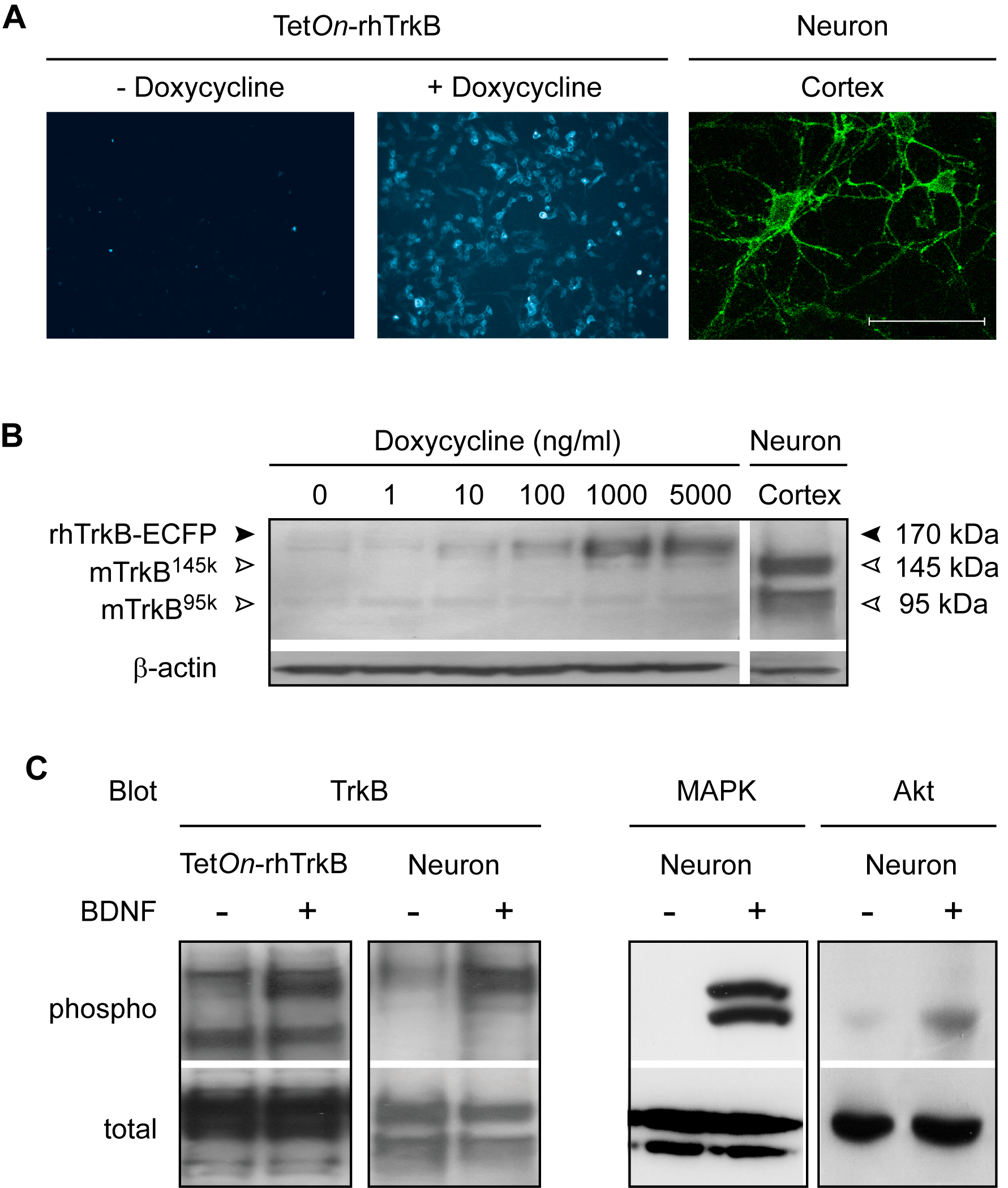

Supplement: Figure S1 — Cells systems for the analysis of recombinant and neuronal TrkB receptors. (A) Representative fluorescence photomicrographs of TetOn-rhTrkB inducible cells following an overnight incubation with (middle panel) or without (left panel) doxycycline and confocal TrkB immunofluorescence in mouse cortical neurons (right panel). Scale bar, 55 µm. (B) Western blot analysis of TrkB expression in TetOn-rhTrkB cells treated with increasing concentrations of doxycycline and in cultured mouse cortical neurons. One band was detected ({similar, tilde operator } 170 kDa, closed arrowhead) for recombinant human TrkB fused to ECFP whereas two distinct bands (95 and 145 kDa, open arrowhead) were revealed for neuronal TrkB. Doxycycline dose-dependently induced the expression of rhTrkB-ECFP in TetOn-rhTrkB cells and was used at the optimal concentration of 1000 ng/ml in all further experiments. (C) Representative western blot analysis of total and phosphorylated TrkB in TetOn-rhTrkB cells and neurons, and total and phosphorylated MAPK and Akt in cortical neurons, after treatment with or without BDNF (1 nM). (2.27 MB TIF) [file pone.0009777.s002.tif]

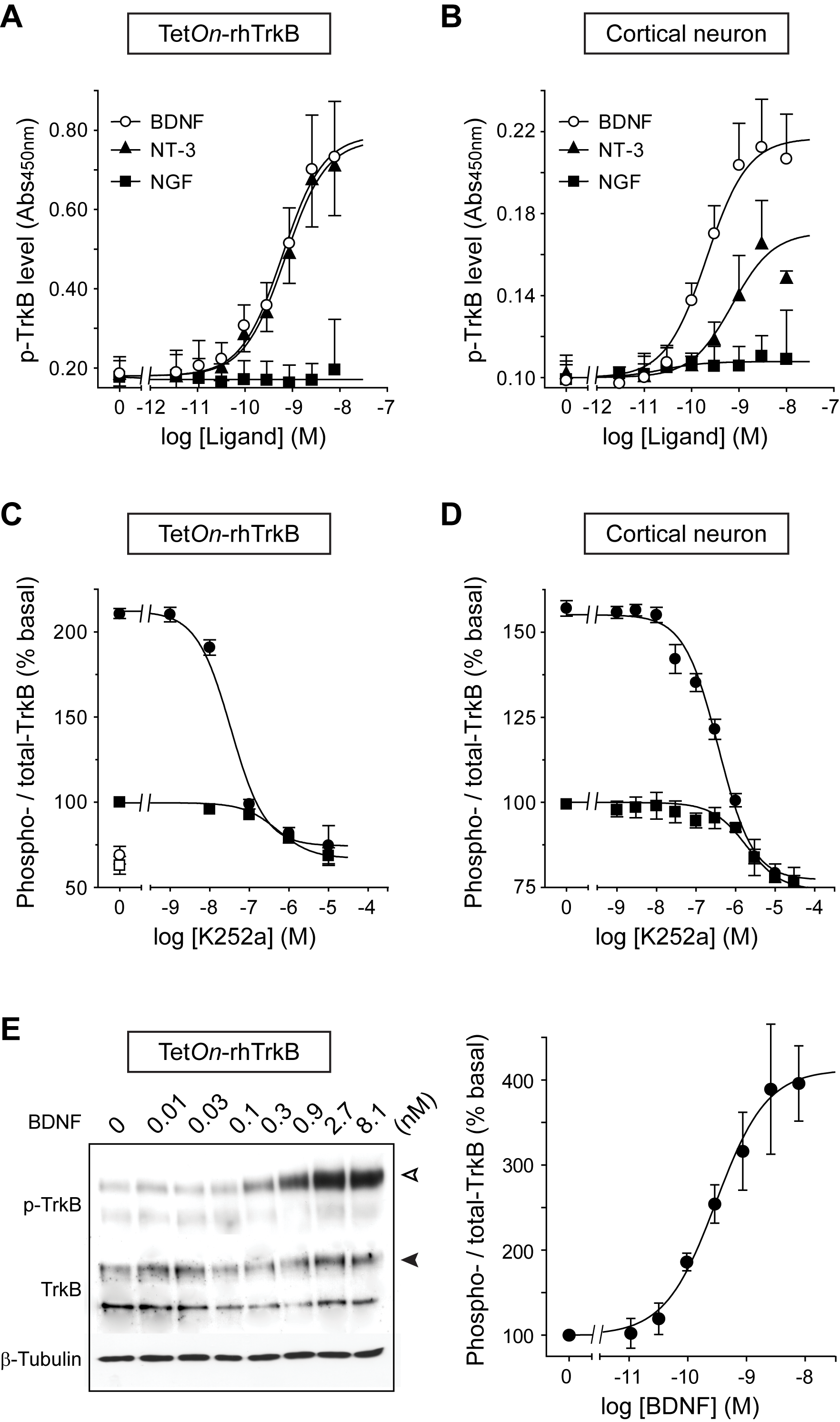

Supplement: Figure S2 — Pharmacology of recombinant and neuronal TrkB receptor using KIRA-ELISA. (A,B) KIRA-ELISA concentration-response curves for BDNF, NT-3 and NGF in TetOn-rhTrkB cells and in cultured cortical neurons. Results are expressed as mean ± s.e.m. of raw absorbance read at 450 nm in six independent experiments performed in triplicate. (C,D) Inhibition of TrkB phosphorylation by K252a in both TetOn-rhTrkB cells and cortical neurons. Cells were treated for 20 min with K252a prior to treatment with (closed circle) or without (closed square) BDNF (4 nM). K252a was dissolved in dimethyl sulphoxide (DMSO); we verified that DMSO did not affect TrkB activity. As values obtained with 10 µM K252a are not different from those obtained in non-induced TetOn-rhTrkB cells (with BDNF, open circle; without BDNF, open square; C), background signal was defined as the value obtained with 10 µM K252a in KIRA-ELISA studies in both cell types. Data are mean ± s.e.m. of four experiments performed in triplicate and results are expressed as a ratio between phospho-TrkB and total TrkB in percentage of basal. (E) Western blot analysis of concentration-response curve for BDNF in TetOn-rhTrkB cells. Representative blots are shown for phospho-TrkB, TrkB and β-Tubulin (left) as well as quantitative analysis of band intensity (right). White and black arrows show the active form of TrkB. Data are mean ± s.e.m. of three experiments and results are expressed as a ratio between phospho-TrkB and total TrkB in percentage of basal. (1.05 MB TIF) [file pone.0009777.s003.tif]

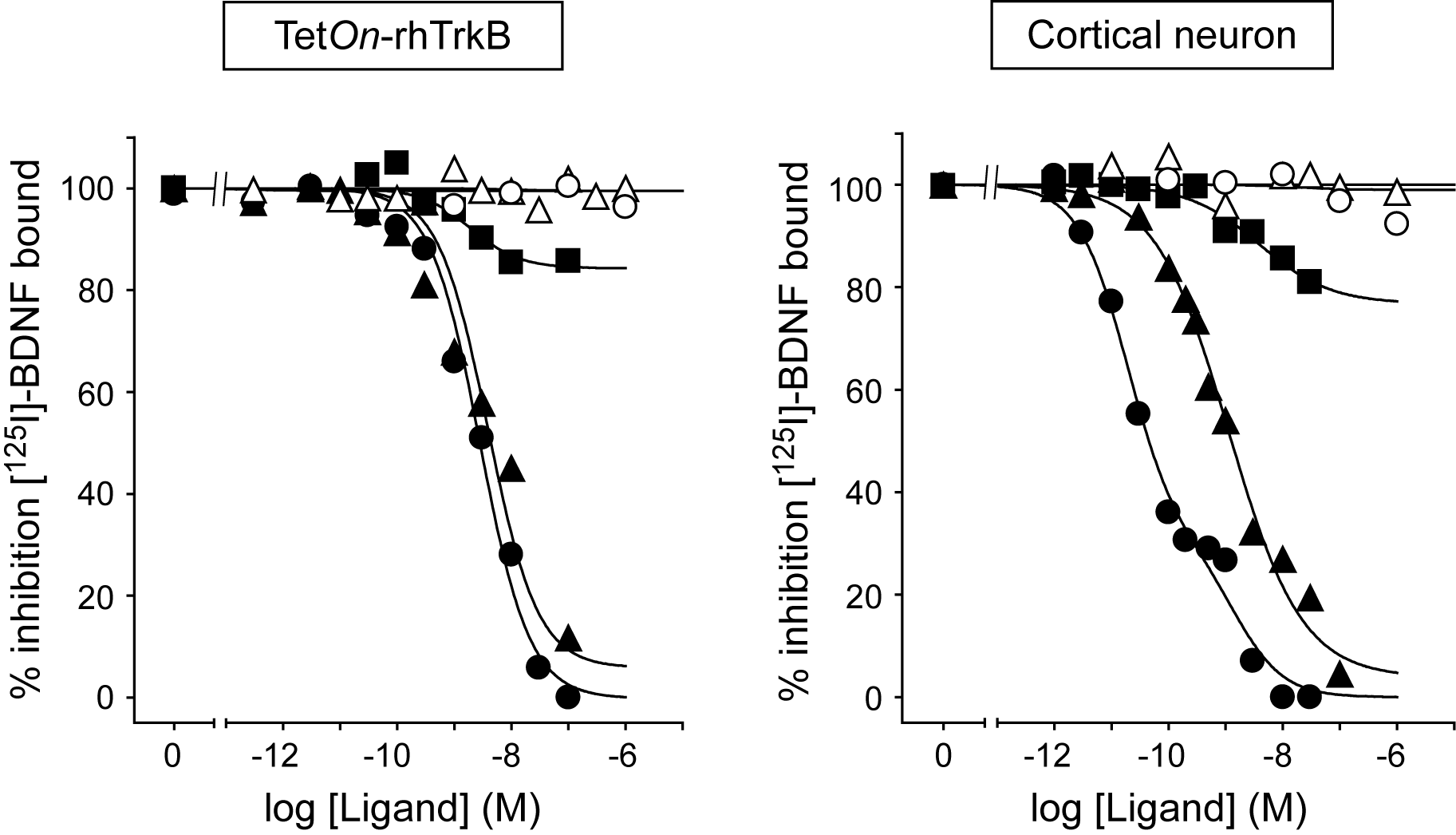

Supplement: Figure S3 — Cyclotraxin-B does not compete with [125I]-BDNF binding to recombinant nor to neuronal TrkB receptors. Effect of unlabeled BDNF (closed circle), NT-3 (closed triangle), NGF (closed square), peptide L2-8 (open triangle) and cyclotraxin-B (open circle) on [125I]-BDNF binding to recombinant and neuronal TrkB receptors. Adherent cells were pre-incubated 60 min at 4°C with increasing concentrations of unlabeled BDNF, NT-3, NGF, peptide L2-8 and cyclotraxin-B before an additional 2 hours incubation at 4°C with 200 pM [125I]-BDNF. Each point represents the results from two independent experiments performed in triplicate. (0.25 MB TIF) [file pone.0009777.s004.tif]

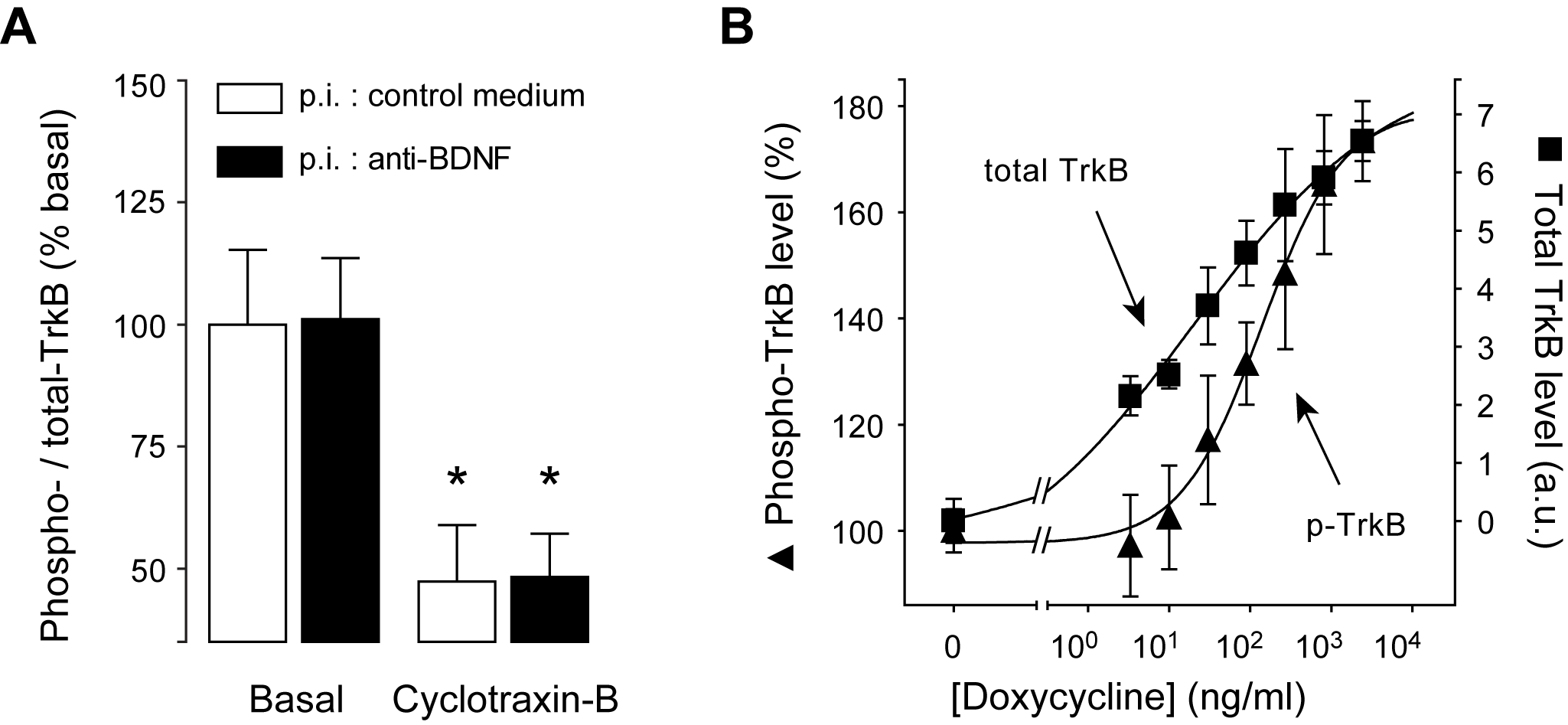

Supplement: Figure S4 — Basal activity of TrkB receptors in absence of BDNF. (A) Absence of endogenous BDNF in cultured cortical neurons. Neuronal cells were incubated 24 hours with a neutralizing anti-BDNF antibody before treatment with cyclotraxin-B. KIRA-ELISA analysis revealed no significant difference in cyclotraxin-B inhibition with or without pretreatment with anti-BDNF. *P<0.01 compared to their respective basal condition. Data are mean ± s.e.m. (triplicates, n = 3). p.i., preincubation. (B) BDNF-independent TrkB activity depends on TrkB density in TetOn-rhTrkB cells. Cells were incubated overnight with increasing concentrations of doxycycline. Total and phospho-TrkB were then evaluated using fluorescence quantification and KIRA-ELISA, respectively. (0.26 MB TIF) [file pone.0009777.s005.tif]

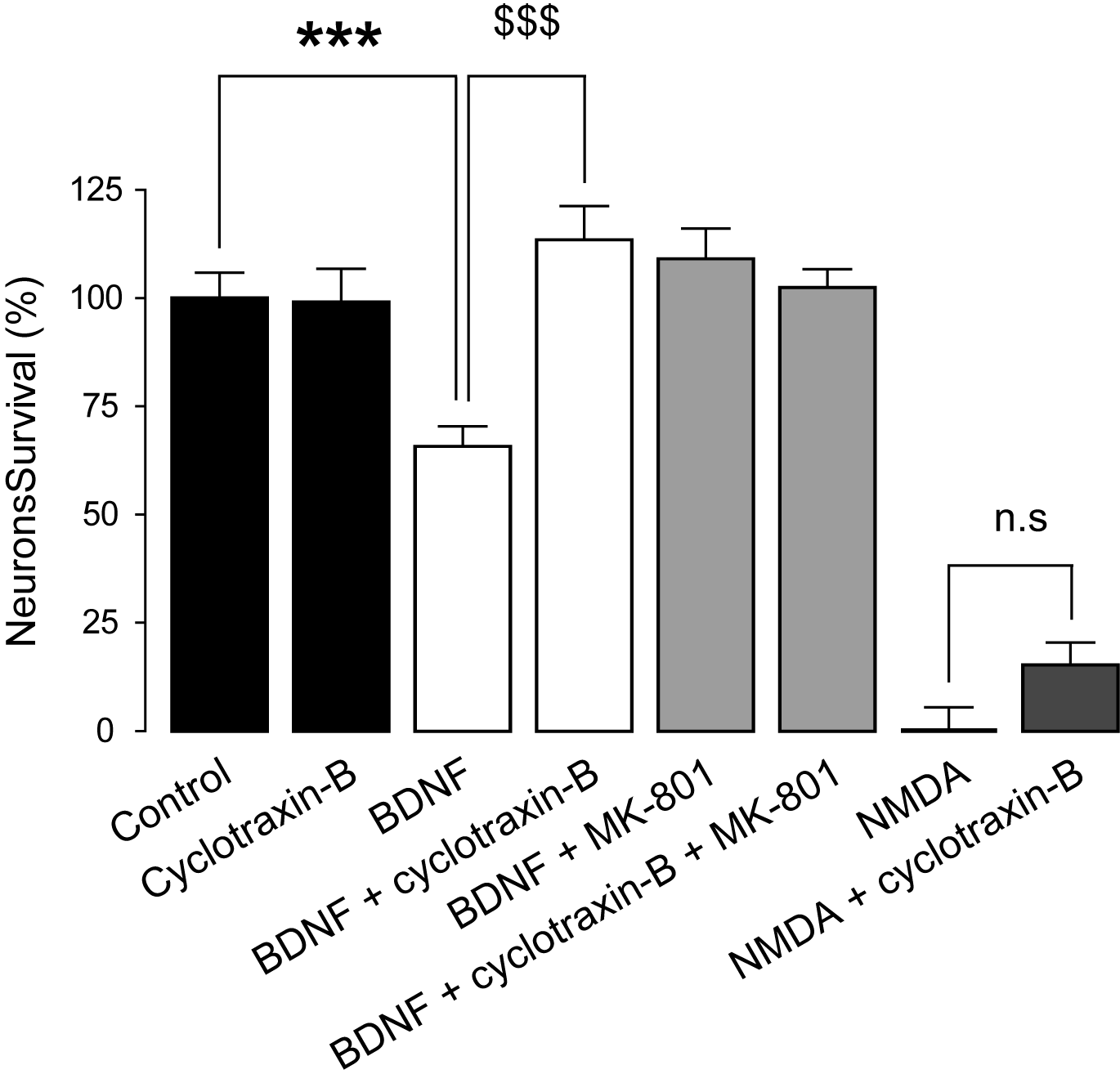

Supplement: Figure S5 — BDNF-induced neuronal necrosis is prevented by cyclotraxin-B. Cyclotraxin-B prevents BDNF-induced neurons death through a NMDA-independent pathway. Cortical neurons were treated with cyclotraxin-B (200 nM), BDNF (4 nM), NMDA (200 µM) or MK-801 (1 µM), as indicated. Data are mean ± s.e.m. (octuples, n = 6) expressed in percentage of control. ***P<0.001 compared to control; $$$P<0.001, compared to BDNF; n.s, non significant. (0.30 MB TIF) [file pone.0009777.s006.tif]

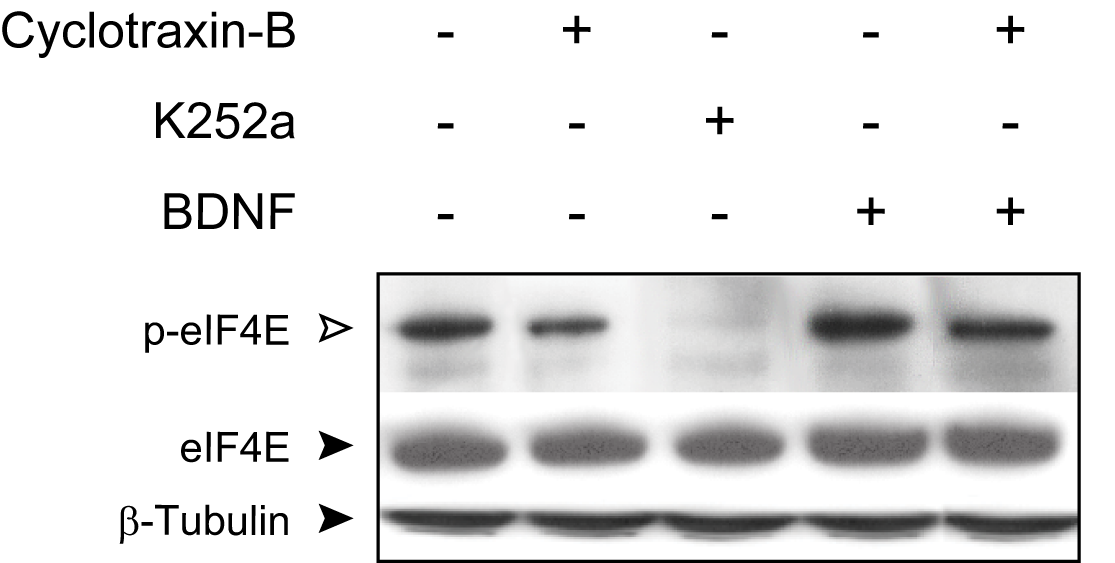

Supplement: Figure S6 — Cyclotraxin-B inhibits eIF4E phosphorylation in cortical neurons. Cortical neurons were exposed to cyclotraxin-B, K252a and BDNF, as indicated. Phosphorylation of eIF4E was quantified by immunoblots using anti-phospho and anti-total-eIF4E antibodies. A representative western-blot is shown here. (0.33 MB TIF) [file pone.0009777.s007.tif]

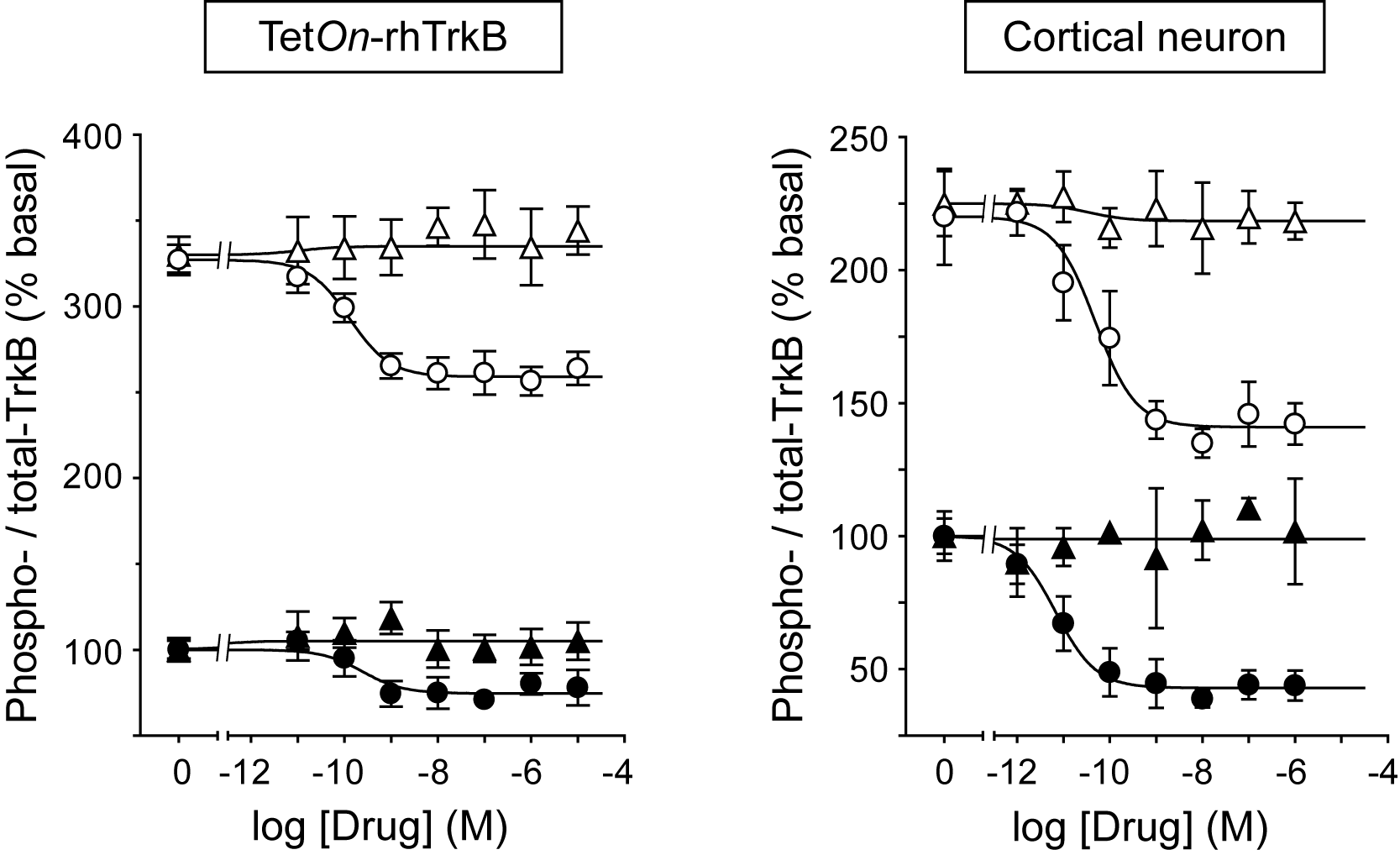

Supplement: Figure S7 — Tat-cyclotraxin-B inhibits both recombinant and neuronal TrkB receptors similarly to cyclotraxin-B. Characterization of TrkB inhibition by tat-cyclotraxin-B using KIRA-ELISA assays in TetOn-rhTrkB cells and in cortical neurons. Increasing concentrations of tat-empty and tat-cyclotraxin-B were added to the cells for 30 min prior to treatment with or without 4 nM BDNF for 20 min (tat-cyclotraxin-B, closed circle; tat-cyclotraxin-B + BDNF, open circle; tat-empty, closed triangle; tat-empty + BDNF, open triangle). Data are mean ± s.e.m. (triplicates, n = 4, TetOn-rhTrkB; n = 2, cortical neurons). (0.24 MB TIF) [file pone.0009777.s008.tif]

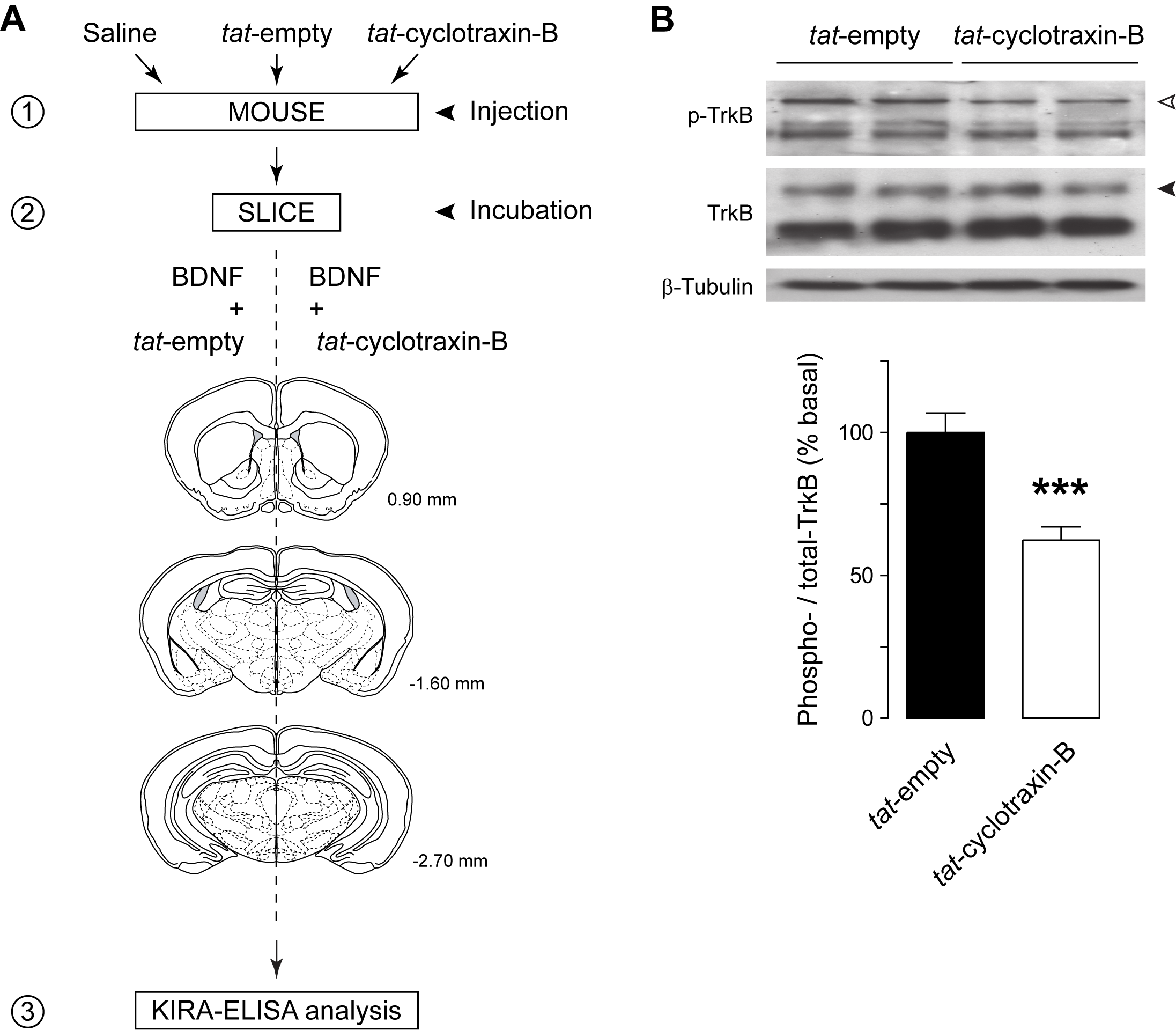

Supplement: Figure S8 — Protocol for in vivo KIRA-ELISA analysis after intravenous injection. (A) Adult mice received two i.v. injections of saline buffer, tat-empty or tat-cyclotraxin-B (1). Brains were then sliced at three levels (bregma 0.90 mm: caudate putamen/nucleus accumbens level; bregma −1.60 mm: dorsal hippocampus level; bregma −2.70 mm: ventral hippocampus level) and each half-slice was treated with BDNF (4 nM) and either tat-empty or tat-cyclotraxin-B (1 µM) (2). Slices were then solubilized and subjected to KIRA-ELISA analyzes (3). (B) Western blot analysis of tat-cyclotraxin-B effect on TrkB activation in vivo. Concentration-response curve for BDNF in TetOn-rhTrkB cells. Representative western blots of brain phospho-TrkB, total-TrkB and β-Tubulin from mice injected with either tat-empty or tat-cyclotraxin-B are shown (up). White and black arrows show the active form of TrkB. Bands intensity have been quantified (down) Data are mean ± s.e.m. (duplicates, n = 6 mice/group) expressed in percentage of control. ***P<0.001 compared to control. (0.95 MB TIF) [file pone.0009777.s009.tif]
